# The putative endo-1,4-β-D-glucanase GLU3 regulates cellulose biosynthesis in barley roots

**DOI:** 10.1093/plphys/kiaf311

**Published:** 2025-07-17

**Authors:** Li Guo, Serena Rosignoli, Magnus Wohlfahrt Rasmussen, Kiran Suresh, Giuseppe Sangiorgi, Francesco Camerlengo, Viktoria V Zeisler-Diehl, Lukas Schreiber, Christoph Dockter, Markus Pauly, Roberto Tuberosa, Frank Hochholdinger, Silvio Salvi

**Affiliations:** Institute of Crop Science and Resource Conservation, Department of Crop Functional Genomics, University of Bonn, Bonn 53117, Germany; Department of Agricultural and Food Sciences, University of Bologna, Bologna 40127, Italy; Carlsberg Research Laboratory, J.C. Jacobsens Gade 4, Copenhagen V DK-1799, Denmark; Institute for Cellular and Molecular Botany, Department of Ecophysiology, University of Bonn, Bonn 53115, Germany; Department of Agricultural and Food Sciences, University of Bologna, Bologna 40127, Italy; Department of Agricultural and Food Sciences, University of Bologna, Bologna 40127, Italy; Institute for Cellular and Molecular Botany, Department of Ecophysiology, University of Bonn, Bonn 53115, Germany; Institute for Cellular and Molecular Botany, Department of Ecophysiology, University of Bonn, Bonn 53115, Germany; Carlsberg Research Laboratory, J.C. Jacobsens Gade 4, Copenhagen V DK-1799, Denmark; Institute of Plant Cell Biology and Biotechnology, Heinrich Heine University, Düsseldorf 40225, Germany; Department of Agricultural and Food Sciences, University of Bologna, Bologna 40127, Italy; Institute of Crop Science and Resource Conservation, Department of Crop Functional Genomics, University of Bonn, Bonn 53117, Germany; Department of Agricultural and Food Sciences, University of Bologna, Bologna 40127, Italy

## Abstract

The plant cell wall is a crucial structure that ensures plant cell integrity and facilitates environmental adaptation. Cellulose is the primary component of the plant cell wall. Its biosynthesis is orchestrated through the plasma membrane-localized multiprotein cellulose synthase complex, which includes a membrane-anchored endo-1,4-ß-glucanase. Here, we identified a barley (*Hordeum vulgare*) mutant with short roots resulting from repressed cell division and elongation, which we designated *H. vulgare endo-β-1,4-D-glucanase 3-1* (*hvglu3-1*). *HvGLU3* encodes a putative membrane-anchored endo-1,4-ß-glucanase that is highly conserved across plant species. The *hvglu3-1* mutant exhibited a 60% reduction in cellulose content, accompanied by changes in hemicellulose and suberin levels and an altered lignin structure in the roots. Subcellular localization analyses and bimolecular fluorescence complementation assays suggested a direct interaction between HvGLU3 and primary cellulose synthases. We investigated the reprogramming of the tissue-specific transcriptome in *hvglu3-1* root tips using a combination of laser capture microdissection and RNA sequencing. This approach revealed that 74% of all genes that are actively expressed in the elongation zone are influenced by root cellulose biosynthesis. Gene coexpression analyses highlighted the essential role of cellulose biosynthesis in diverse biological processes, including cell wall organization, phytohormone signaling, and stress responses, to regulate root tissue development. Overall, our study demonstrates the partially conserved role of HvGLU3 in controlling cellulose biosynthesis in roots and provides valuable transcriptomic resources for future studies.

## Teaser

The putative endo-1,4-β-D-glucanase GLU3 controls barley root development by regulating cellulose biosynthesis and modifying cell wall composition.

## Introduction

The cell wall provides a framework to support the cell structure, protects against infection by pathogens, and mediates the communication between cells to regulate plant development (Houston et al. 2016; Zhang et al. 2021). Due to the diverse roles of the cell wall, its composition and organization are variable among tissue types and plant species (Hoffmann et al. 2021; Zhang et al. 2021).

In general, plants produce a multilayered cell wall with a middle lamella and a dynamic primary cell wall in young cells. As cells mature, specialized cells develop an additional secondary cell wall inside the primary cell wall (Houston et al. 2016; Kumar et al. 2016). The middle lamella, primarily composed of pectin, is located between the primary cell walls of neighboring cells and thus ensures adhesion of adjacent cells (Zamil and Geitmann 2017). In growing plant cells, the primary cell wall is located between the middle lamella and the plasma membrane, which is hydrated, allowing metabolic polymer rearrangements relatively and hence facilitating cell expansion (Houston et al. 2016; Pękala et al. 2023). The secondary cell wall, typically thicker, hydrophobic, and more rigid than the primary cell wall, offers additional protection and structural support to fully grown mature cells (Kumar et al. 2016; Xu et al. 2022). The primary cell wall consists mainly of cellulose, hemicelluloses, and pectin, whereas the secondary cell wall is mainly composed of cellulose, hemicellulose, and lignin (Cosgrove and Jarvis 2012; Kumar et al. 2016; Zhong et al. 2019; Xu et al. 2022). Given the critical functions of the cell wall in plant development, any disturbance in its organization or composition likely results in aberrant development outcomes.

Cellulose, as the most prominent constituent of the plant cell wall, is a complex carbohydrate chain consisting of up to 14,000 glucose molecules linked together (Somerville et al. 2004). Cellulose is synthesized by cellulose synthase (CESA) complexes (CSCs), which are commonly composed of hexametric symmetrical rosettes, with each rosette comprising CESA trimers (Hill et al. 2015; Polko and Kieber 2019). In *Arabidopsis* (*Arabidopsis thaliana*), primary and secondary cell wall cellulose biosynthesis is facilitated by different CESAs (Li et al. 2014; Polko and Kieber 2019; Pedersen et al. 2023). CSCs are assembled in the Golgi apparatus and then transported to the plasma membrane, where they are presumably activated to initiate the biosynthesis of cellulose (Li et al. 2014; Polko and Kieber 2019; Pedersen et al. 2023). The coordination of CESA synthesis, CSC assembly, delivery, and catalytic activity involves intricate interactions with numerous accessory proteins, including KORRIGAN1 (KOR1) in *Arabidopsis* (Lampugnani et al. 2019; Pedersen et al. 2023). Heterologous expression of the catalytic domain of KOR exhibited endo-1,4-ß-glucanase activity (Mølhøj et al. 2001), and hence KOR1 represents a putative membrane-anchored endo-1,4-ß-glucanase, an enzyme present in diverse plant species (Buchanan et al. 2012; Perrot et al. 2022). Barley (*Hordeum vulgare*) encodes at least 22 endo-1,4-ß-glucanases, none of which have been functionally characterized (Buchanan et al. 2012).

In this study, we identified a barley short root mutant, *H. vulgare endo-β-1,4-D-glucanase 3-1* (*hvglu3-1*), by screening a barley Targeting Induced Local Lesions in Genomes (TILLING) mutant collection (Talamè et al. 2008). Employing a combination of MutMap+ (Fekih et al. 2013) and variant calling, we cloned *HvGLU3* (*HORVU.MOREX.r3.2HG0178520.1*), which encodes a putative membrane-anchored endo-1,4-ß-glucanase. Histological characterization of *hvglu3-1* revealed effects of the gene on root cell wall composition achieved through the regulation of cellulose biosynthesis. Through bimolecular fluorescence complementation (BiFC) assay, we demonstrated direct interaction between HvGLU3 and primary CESAs. Furthermore, tissue-specific RNA sequencing (RNA-seq) and gene coexpression network analyses highlighted the central involvement of cellulose biosynthesis mediated by HvGLU3 in regulating root tissue development, particularly in root cap and root epidermis development.

## Results

### The *hvglu3-1* mutant displays short shoot and roots with severely disorganized root surface

**Figure 1.**
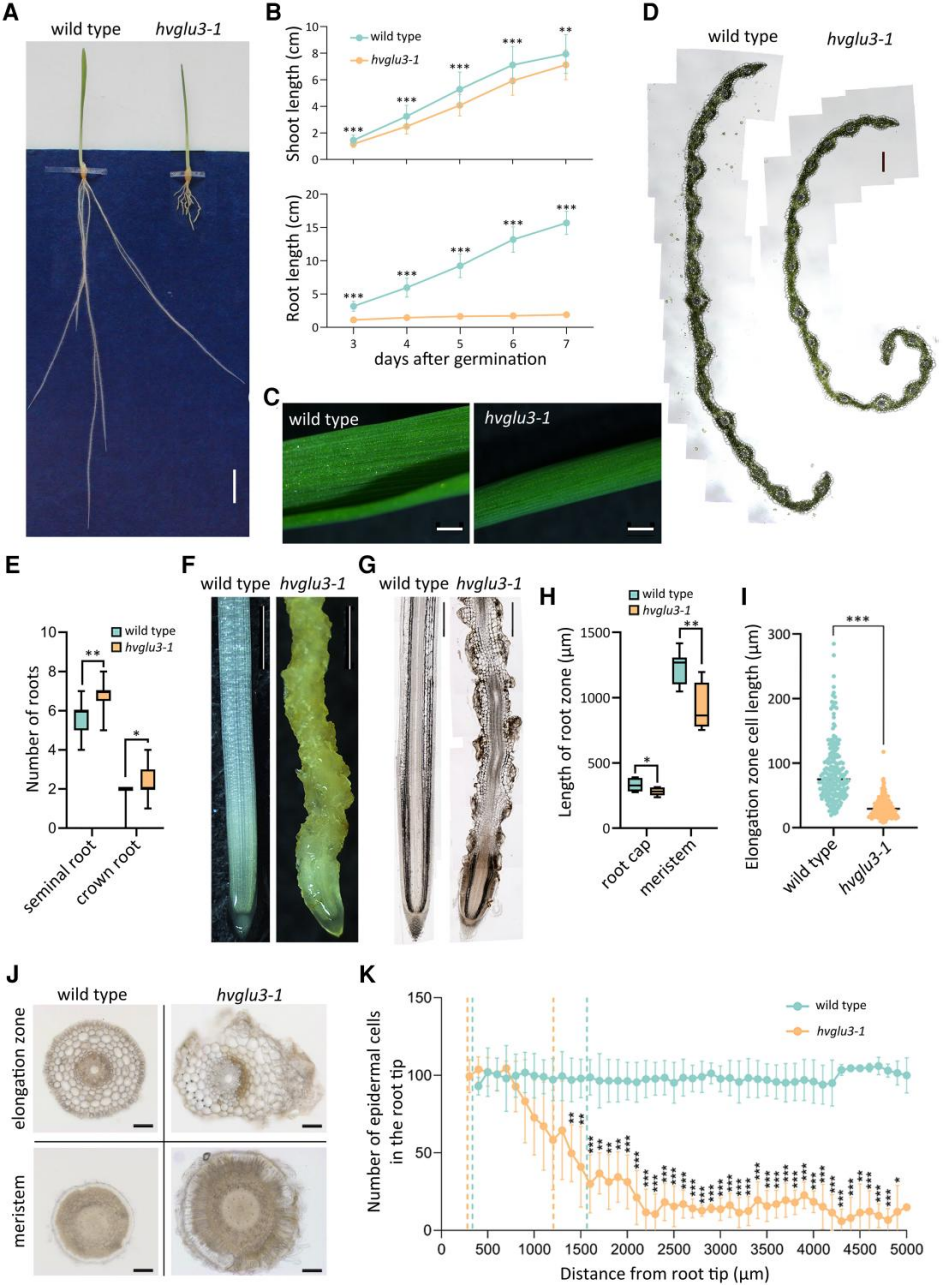
Phenotype of the mutant *hvglu3-1*. **A)** Representative picture of 7-d-old (days after germination) WT and *hvglu3-1* mutant seedlings grown on 2D germination paper. Scale bar = 1 cm. **B)** Shoot (upper) and seminal root length (lower) of WT and *hvglu3-1* from 3 to 7 d after germination. *n* = 10 seedlings per genotype with 6 to 7 roots measured per seedling. Two-tailed *t*-test. **P < 0.01; ***P < 0.001. Sd is depicted. **C)** Stereo microscopic observation of 7-d-old leaves of WT and *hvglu3-1*. The region below 5 mm from the leaf tip is shown. Scale bar = 1 mm. **D)** Cross section from the leaf zone is shown in **C)**. These images are composite images. Scale bar for both images = 200 *µ*m. **E)** Seminal roots and crown root number of 20-d-old WT and *hvglu3-1* plants. *n* = 8 for WT and *n* = 5 the *hvglu3-1*. Two-tailed *t*-test. *P < 0.05; **P < 0.01. **F)** Stereo microscopic observation of 7-d-old seminal roots of WT and *hvglu3-1*. Scale bar = 1.5 mm. **G)** Longitudinal sections of 7-d-old WT and *hvglu3-1* seminal roots. Scale bar = 0.5 mm. These images are composite images. **H)** Length of the root cap and the root meristem of 7-d-old WT and *hvglu3-1* seminal roots. *n* = 7 for each genotype. Two-tailed *t*-test. *P < 0.05; **P < 0.01. In the boxplots **E)** and **H)**, the center line within each box represents the median; box limits indicate the upper and lower quartiles; the whiskers extend to the minimum and maximum values. **I)** Length of the first 28 cells in the outmost files of the cortex in 7-d-old WT and *hvglu3-1* seminal roots. Cells from 2 files at both sides were measured per root; 3 roots were measured per genotype. The dots represent individual data point and the center line indicates the mean value. Two-tailed *t*-test. ***P < 0.001. **J)** Cross sections of 7-d-old WT and *hvglu3-1* seminal roots at 1 mm (meristem) and 1.8 mm (elongation zone) from the root tip. Scale bar = 100 *µ*m. **K)** Number of epidermal cells in the first 0.5 mm segment of 7-d-old WT and *hvglu3-1* seminal roots. Root segments before the first dashed line represent the root cap, while the root segments between 2 dashed lines of the same color indicate the meristem. Cell numbers of sections equidistant to the root tip were compared between WT and *hvglu3-1*. *n* = 4 per genotype. Sd is depicted. Two-tailed *t*-test. *P < 0.05; **P < 0.01; ***P < 0.001.

To identify genes regulating root development, we phenotypically screened a sodium azide-mutagenized barley population (Talamè et al. 2008) and identified a mutant (Line TM390) with significantly shorter shoot and seminal roots (Fig. 1, A and B), which we designated *hvglu3-1*. The reduced root elongation of the *hvglu3-1* mutant became more pronounced as the seedling continued to grow (Fig. 1B). Despite the delayed shoot development, the mutant exhibited normal leaf morphology and structure comparable to the wild type (WT; Fig. 1, C and D). Compared with the WT, the *hvglu3-1* mutant produced more seminal roots and crown roots (Fig. 1E). Notably, the mutant root displayed a twisted shape with a rough, yellowish surface above the root cap (Fig. 1F). In longitudinal orientation, the *hvglu3-1* mutant roots showed a marked loss of epidermal cells (Fig. 1G) and had a significantly shorter root cap and root meristem compared to the WT (Fig. 1H). Moreover, cells at the distal elongation zone were significantly longer in WT than in the mutant (Fig. 1I). To investigate whether cell activity was affected in mutant *hvglu3-1* roots, we stained the roots with Evans blue. The mutant roots displayed a noticeably darker staining compared with WT roots (Supplementary Fig. S1, A and B), indicating increased cell damage. A subsequent triphenyltetrazolium chloride assay revealed a significantly higher level of cell metabolic activity in mutant *hvglu3-1* roots compared with WT roots (Supplementary Fig. S1C).

To examine cellular organization in the mutant *hvglu3-1* roots, we prepared cross sections of WT and *hvglu3-1* seminal roots (Fig. 1J). Results revealed that the radial pattern was not disturbed in the mutant roots (Fig. 1J). However, we observed a significantly thicker epidermis in the meristem, accompanied by a substantial loss of epidermal cells in the elongation zone of mutant *hvglu3-1* roots (Fig. 1J). The thicker root epidermis was evident as early as the embryonic development stage (Supplementary Fig. S1D). Using Direct Red 23 staining, we observed that the epidermal cells in the meristem of the *hvglu3-1* mutant exhibited intact cell walls (Supplementary Fig. S1E).

To better characterize the phenotypical defects of the mutant *hvglu3-1*, we conducted a series of sections of 0.5 cm segments form the root tip, middle, and base of WT and mutant roots (Supplementary Fig. S2A). Compared with WT, we observed a dramatically larger section area and epidermal area in the meristem of the mutant *hvglu3-1* (Supplementary Fig. S2B). These differences diminished in the apical elongation zone, coinciding with a sharp reduction of the number of epidermal cells starting in this zone in mutant *hvglu3-1* roots (Fig. 1K).

We next investigated the crown root phenotype in WT and mutant *hvglu3-1*. Compared with WT, crown roots of the mutant *hvglu3-1* formed earlier and displayed defective phenotypes similar to those observed in seminal roots (Supplementary Fig. S3, A to C).

The root defects persisted throughout the entire lifespan of the mutant *hvglu3-1* (Supplementary Fig. S3D), leading to impaired aboveground development (Supplementary Fig. S3E), including shorter shoots at flowering stage, fewer tillers, and smaller leaf area (Supplementary Fig. S3, F to H).

### *HvGLU3* encodes an endo-1,4-ß-glucanase

**Figure 2.**
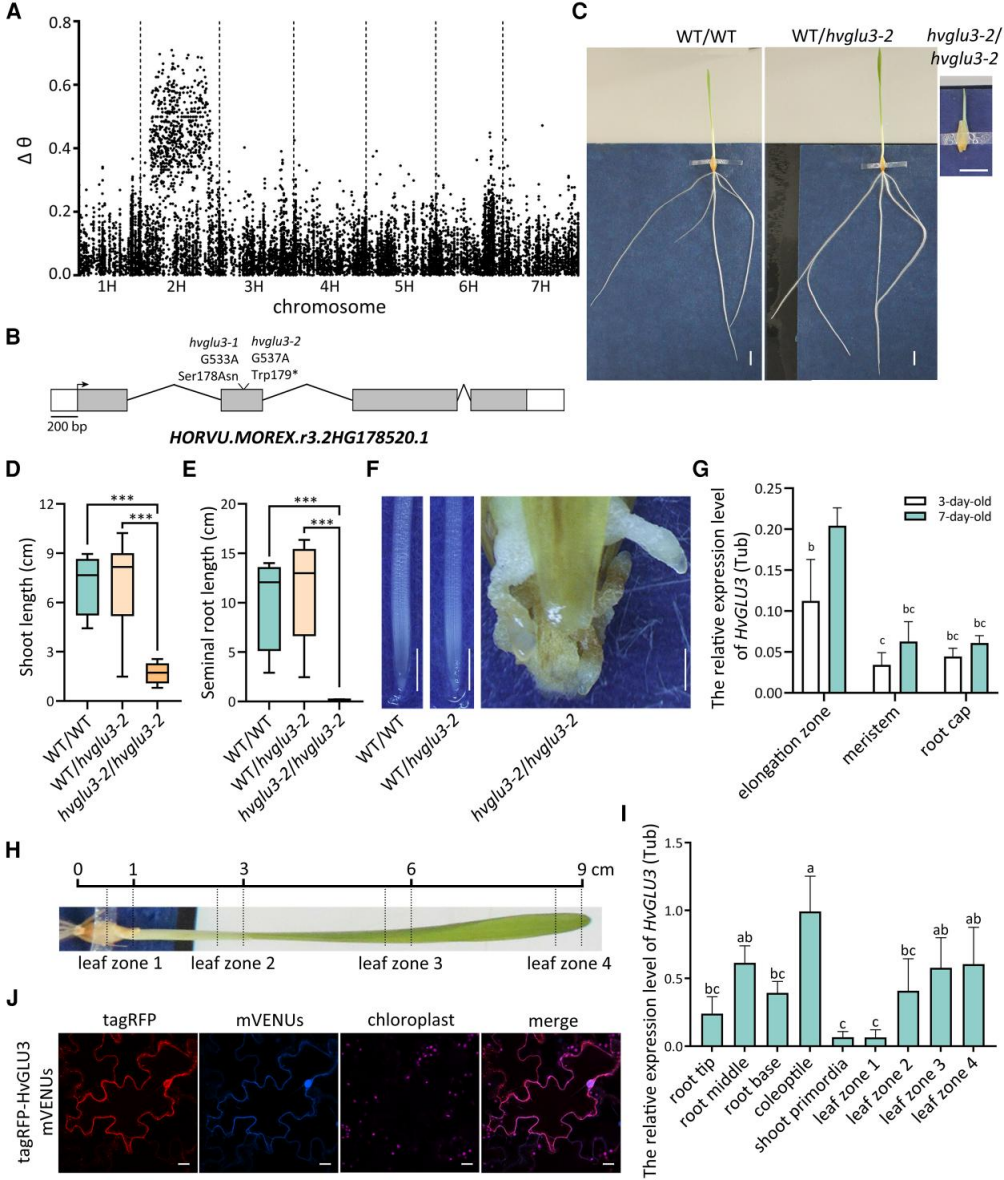
Cloning of the gene responsible for root phenotype of the mutant *hvglu3-1*. **A)** Association of SNP markers with the phenotype of the mutant *hvglu3-1* across the barley genome. The chart shows the value of DSNP index as a function of chromosomic position. Chromosome 2H has a strong signal in the interval between 75 and 610 Mb. DSNP index is calculated as the difference between the SNP index of the mutant bulk and that of the WT bulk. SNP index is the ratio between the number of variant reads and read depth at a position. **B)** Gene structure of *HvGLU3* (*HORVU.MOREX.r3.2HG0178520.1*). Arrow represents the translational start site, white boxes depict UTRs, exons are depicted as gray boxes, and introns are depicted by lines. The mutations in *hvglu3-1* (G533A) and *hvglu3-2* (G537A) are indicated. **C)** Representative picture of 7-d-old WT (WT/WT), heterozygous (WT/*hvglu3-2*) and homozygous (*hvglu3-2*/*hvglu3-2*) *hvglu3-2* mutant seedlings. Scale bar = 1 cm. **D)** Shoot length of 7-d-old WT/WT, WT/*hvglu3-2*, and *hvglu3-2*/*hvglu3-2* seedlings. **E)** Seminal root length of 7-d-old WT/WT, WT/*hvglu3-2*, and *hvglu3-2*/*hvglu3-2* seedlings. The average length of all roots of each seedling was calculated. **D**, **E)**  *n* = 4 for WT/WT and *hvglu3-2*/*hvglu3-2*, *n* = 10 for WT/*hvglu3-2*. In the boxplots, the center line within each box represents the median; box limits indicate the upper and lower quartiles; the whiskers extend to the minimum and maximum values. Two-tailed *t*-test. **P < 0.01; ***P < 0.001. **F)** Exemplary picture of 7-d-old seminal root tips of WT/WT, WT/*hvglu3-2*, and *hvglu3-2*/*hvglu3-2* seedlings. Scale bar = 1 mm. **G)** The expression of *HvGLU3* in the indicated root zones of 3-d-old and 7-d-old WT seminal roots. The root segments from 4 roots were collected per plant. Samples from 5 seedlings were pooled as 1 biological replicate. Four biological replicates were used for all zones. **H)** Examples of leaf zones in which the expression level of *HvGLU3* has been determined in **I)**. **I)** The expression of *HvGLU3* in the indicated tissues of 7-d-old WT seedlings. Samples from 4 seedlings were pooled as 1 biological replicate. Four biological replicates were used for all tissues. **G**, **I)** Statistically significant differences among groups were analyzed using the Lsd test with Bonferroni correction at a significance level of *P* < 0.05. Sd is depicted. **J)** Subcellular localization assay of HvGLU3. tagRFP, red fluorescence; mVenus, yellow fluorescence; chloroplasts, autofluorescence of chloroplasts. Scale bar = 20 *µ*m.

We mapped the causal gene of *hvglu3-1* with MutMap+ applied to plants derived from self-pollination of heterozygous (*HvGLU3-1/hvglu3-1*) plants. After whole genome sequencing of the 2 bulks, DSNP index was calculated for each variant, and the resulting 140,298 values were plotted against the chromosomic position (Fig. 2A). Chromosome 2H has a broad interval of 535 Mb between 75 and 610 Mb where DSNP index is >0.5 (Fig. 2A). No such peak region was found in other chromosomes. Therefore, *HvGLU3* maps on chromosome 2H in the region between 75 and 610 Mb, which overlaps with the low recombinogenic pericentromeric region of barley 2H chromosome.

Filtering the *HvGLU3* single nucleotide polymorphisms (SNPs) with the results of variant calling on the 2 bulks, we found only 5 candidate genes for the whole genome, in which all of them are in chromosome 2H, in accordance with the interval produced by MutMap+. After amplifying and sequencing the 5 genes in 100 mutant seedlings, we discovered a complete association between the phenotype and a mutation in the *HORVU.MOREX.r3.2HG0178520.1* (*HvGLU3*) gene. This gene encodes a putative endo-1,4-ß-glucanase. A single nucleotide substitution (G533A) in the second exon of the gene resulted in an amino acid change (Ser^178^ to Asn) in the protein (Fig. 2B). The sequence of this protein displays high conservation across various species, and the mutation site in the mutant *hvglu3-1* is conserved among its homologs (Supplementary Fig. S4A).

To validate that the root phenotype of the mutant *hvglu3-1* was due to the point mutation in the *HvGLU3* gene, we isolated an additional mutant allele, *hvglu3-2*, from the FIND-IT barley collection (Knudsen et al. 2022). A single nucleotide substitution (G537A) was introduced into the *HvGLU3* gene in the *hvglu3-2* mutant, leading to a premature stop codon (Trp^179^ to stop codon) in the protein (Fig. 2B; Supplementary Fig. S4A). Due to the inviability of the mutant in the later stages of development, we phenotyped and genotyped the progeny of a segregating heterozygous line. We determined significantly shorter shoots and seminal root in the homozygous mutant seedlings compared with the WT and heterozygous seedlings (Fig. 2, C to E). Moreover, an aberrant epidermis and twisted roots were only observed in the homozygous mutant seedlings (Fig. 2F). The similarity of the *hvglu3-1* and *hvglu3-2* mutant phenotypes confirmed that the root phenotype of both mutants was caused by mutations in the *HvGLU3* gene.

We carried out a qPCR analysis to survey the expression of *HvGLU3* in the root cap, the meristem, and the elongation zone of 3-d-old and 7-d-old WT roots. A higher expression level of the *HvGLU3* gene was detected in root zones of 7-d-old roots compared with 3-d-old roots. At both time points, *HvGLU3* displayed the highest expression level in the elongation zone compared with the other 2 root zones (Fig. 2G). Additionally, we examined the expression pattern of *HvGLU3* in whole seedlings (Fig. 2, H and I). The highest expression of *HvGLU3* was detected in the coleoptile, while lower and comparable expression levels were observed in the root tip, middle, and base segments (Fig. 2I). Among the aboveground tissues, *HvGLU3* exhibited the lowest expression levels in the shoot primordia and the youngest leaf zone (Fig. 2I).

To examine the subcellular localization of HvGLU3, we generated constructs containing tagRFP fused to the N-terminal (tagRFP-HvGLU3) end of HvGLU3 and infiltrated them in *Nicotiana benthamiana* leaves. In infiltrated *N. benthamiana* epidermal cells, the tagRFP-HvGLU3 signal was detected in the plasma membrane and punctate compartments surrounding the plasma membrane and nucleus (Fig. 2J).

### HvGLU3 is highly conserved across barley germplasm and not associated with root length variation

We investigated the *HvGLU3* allelic variation using sequence data of 375 accessions, including wild barley, landraces, and cultivars, generated from the WHEALBI germplasm collection (Bustos-Korts et al. 2019). Forty-eight SNPs were identified across *HvGLU3* genomic sequence. Of these, 17 SNPs located within the coding sequence and all exhibited synonymous mutations, with Ka/Ks = 0, suggesting evolutionary conservation of the HvGLU3 protein (Li et al. 1985; Hurst 2002). *HvGLU3* nucleotide diversity was *π* = 0.0021, a value remarkably lower than estimates obtained for other barley genes (Fricano et al. 2009; Morrell et al. 2014). Analysis of *HvGLU3* haplotypes identified 30 distinct variants, 5 of which had frequencies >5% (Supplementary Fig. S4B). These haplotypes were used for association analysis using a phenotypic dataset of seminal root length collected from the WHEALBI collection; however, no association was detected (Supplementary Fig. S4C).

### HvGLU3 is involved in root cell wall organization

**Figure 3.**
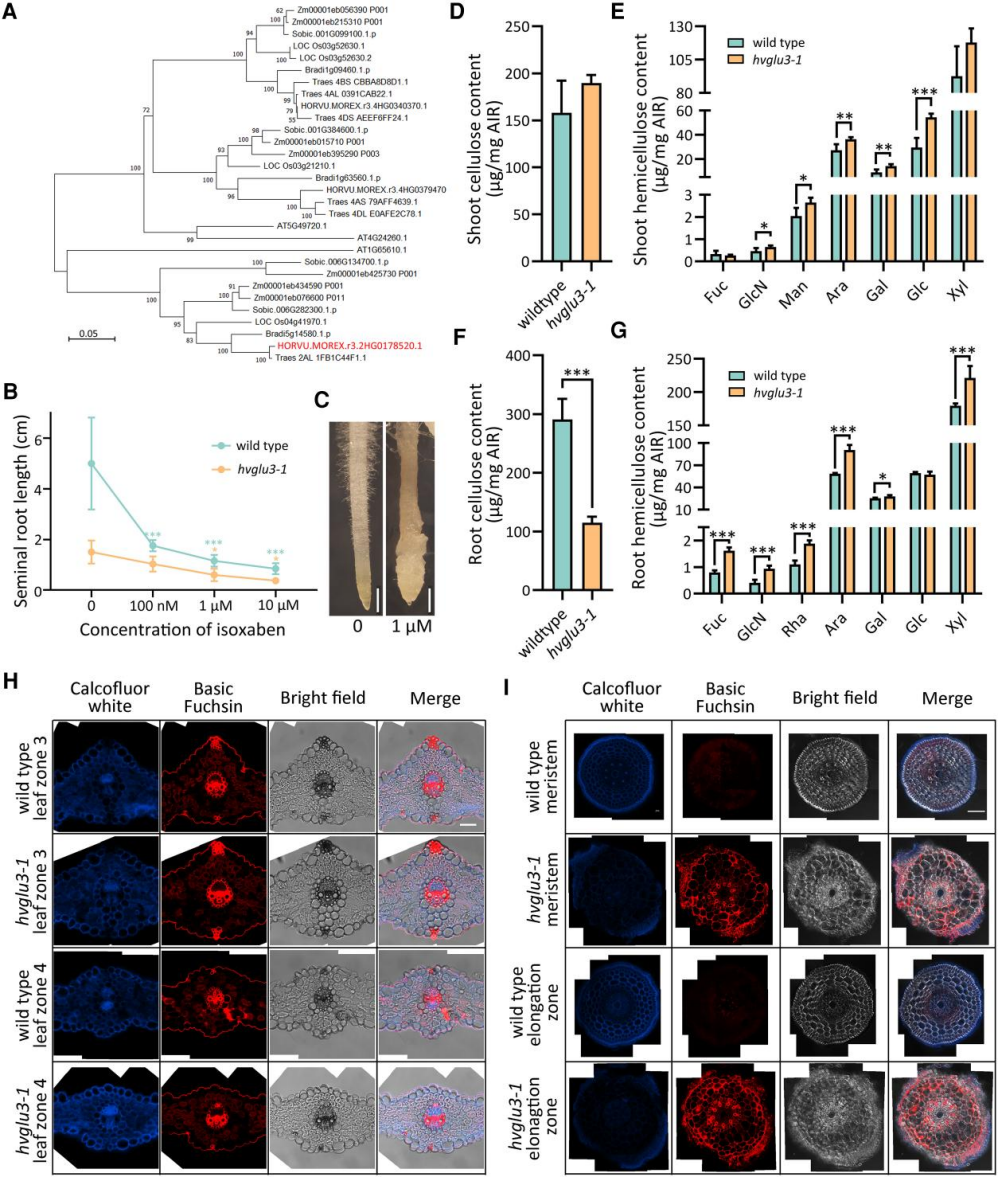
Root cell wall composition was altered in the mutant *hvglu3-1*. **A)** Phylogenetic tree of HvGLU3 and homologous proteins from selected plant species with a sequence identity of >60%. Species abbreviations: Zm, *Zea mays*; Sobic, *Sorghum bicolor*; Os, *Oryza sativa*; Bradi, *Brachypodium distachyon*; Traes, *Triticum aestivum*; HORVU.MOREX, *Hordeum vulgare* (Morex); AT, *Arabidopsis thaliana*. The percentage of replicate trees in which the associated taxa clustered together in the bootstrap test (500 replicates) is shown next to the branches. The branch lengths represent evolutionary distances (number of amino acid substitutions per site) computed using the Poisson correction method. **B)** The seminal root length of 5-d-old WT and mutant (*hvglu3-1*) seedlings grown on solid medium containing the indicated concentration of isoxaben. The root length of seedlings grown on solid medium supplied with isoxaben was compared with the root length of seedlings grown on solid control medium (0). *n* = 8 to 15 per concentration by genotype combination. Sd is depicted. Two-tailed *t*-test. *P < 0.05; ***P < 0.001. **C)** Seminal roots of 5-d-old WT seedlings grown on solid medium without (0) or with 1 *µ*m of isoxaben. Scale bar = 1 mm. **D)** Cellulose content in the shoots of 7-d-old WT and *hvglu3-1*. **E)** Matrix monosaccharide composition in the shoots of 7-d-old WT and *hvglu3-1* seedlings. **F)** Cellulose content of 7-d-old seminal roots of WT and *hvglu3-1*. **G)** Matrix monosaccharide composition in the seminal roots of 7-d-old WT and *hvglu3-1* seedlings. **E**, **G)** Fuc, fucose; GlcN, glucosamine; Rha, rhamnose; Man, mannose; Ara, arabinose; Gal, galactose; Glc, glucose; Xyl, xylose. **D** to **G)** Shoots and all seminal roots from 5 seedlings were collected as 1 replicate; 5 replicates were measured per genotype. AIR, alcohol insoluble residue. Sd is depicted. Two-tailed *t*-test. *P < 0.05; **P < 0.01; ***:*P* < 0.001. **H)** Calcofluor white and basic fuchsin staining for the cross sections from Leaf Zones 3 and 4 of WT and *hvglu3-1*, as shown in Fig. 2H. The images are composite images. Scale bar for all images in this panel = 50 *µ*m. **I)** Calcofluor white and basic fuchsin staining for the sections from the meristem and the elongation zone of WT and *hvglu3-1* seminal roots. The images are composite images. Scale bar for all images in this panel = 100 *µ*m.

Phylogenetic analyses indicated the widespread existence of HvGLU3 homologs throughout the plant kingdom (Fig. 3A). The rice (*Oryza sativa*) ortholog *OsGLU3* (Os04g41970.1) of *HvGLU3* has been demonstrated to be involved in cellulose biosynthesis (Inukai et al. 2012; Zhang et al. 2012). To investigate the role of *HvGLU3* in cellulose biosynthesis, we treated WT and *hvglu3-1* mutant seedlings with isoxaben, a cellulose biosynthesis inhibitor. Isoxaben treatments significantly inhibited the root elongation of WT roots, whereas the inhibitory effect observed in the mutant *hvglu3-1* was weaker (Fig. 3B). Furthermore, application of 1 *µ*m of isoxaben led to a damaged surface of WT roots, which was similar to the rough root surface observed in an untreated *hvglu3-1* mutant (Fig. 3C).

We assessed the impact of *HvGLU3* on the cell wall structure using a number of analytical techniques (Fig. 3, D to G). These analyses revealed that the cellulose content of the wall material of mutant *hvglu3-1* shoots was comparable to that of WT shoots (Fig. 3D). However, the cellulose content in the mutant roots was dramatically reduced to ∼40% of the level observed in WT roots (Fig. 3F). Concomitantly, we observed a higher content of monosaccharides representing matrix polymers in both mutant shoots and roots (Fig. 3, E and G). Among these, elevated levels of arabinose and xylose suggest a higher proportion of the hemicellulose arabinoxylan, while increased rhamnose and galactose indicate altered pectin levels in *hvglu3-1* roots (Fig. 3G). Also, the lignin analysis revealed a higher accumulation of S-type (syringyl [4 hydroxy-3,5-dimethoxyphenyl]) lignin monomers in mutant *hvglu3-1* roots (Supplementary Fig. S5, A to E). Additionally, the amount of aliphatic suberin (acids, ω-OH hydroxyl fatty acid, and diacids) was significantly decreased in mutant *hvglu3-1* roots compared with that in WT roots (Supplementary Fig. S5, F to H). We further investigated the difference in cell wall composition between WT and mutant *hvglu3-1* by histological analyses. No apparent differences were observed in the cellulosic calcofluor white and basic fuchsin signal in stained leaf sections between the 2 genotypes (Fig. 3H; Supplementary Fig. S5I). In contrast, stained root cross sections showed a reduction in cellulosic calcofluor white signal consistent with the wall chemical analysis in the mutant *hvglu3-1* (Fig. 3, F and I). Moreover, we detected a higher basic fuchsin signal in root sections of the mutant *hvglu3-1*, indicating a larger abundance of lignin in those cell walls (Fig. 3I).

### HvGLU3 interacts with primary CESAs

To examine the association between HvGLU3 and CESAs, we first identified CESAs in barley by blasting *Arabidopsis* CESAs in the barley Morex v.3 proteome (Mascher et al. 2021 ). This revealed 8 CESAs in barley, with CESA1, CESA2, CESA3, CESA5, and CESA6 identified as primary CESAs and CESA4, CESA7, and CESA8 as secondary CESAs (Supplementary Fig. S6A). Among them, CESA8 was not included in subsequent studies due to challenges encountered during construct preparation. By infiltrating *N. benthamiana* leaves with constructs containing tagRFP-HvGLU3 and mVenus fused cellulose synthases (mVenus-CESA1/2/3/4/5/6/7), we observed colocalization between HvGLU3 and the primary CESA1, CESA2, CESA3, CESA5, and CESA6 (Supplementary Fig. S6B). However, we did not observe colocalization between HvGLU3 and the secondary CESA4 and CESA7 (Supplementary Fig. S6C).

**Figure 4.**
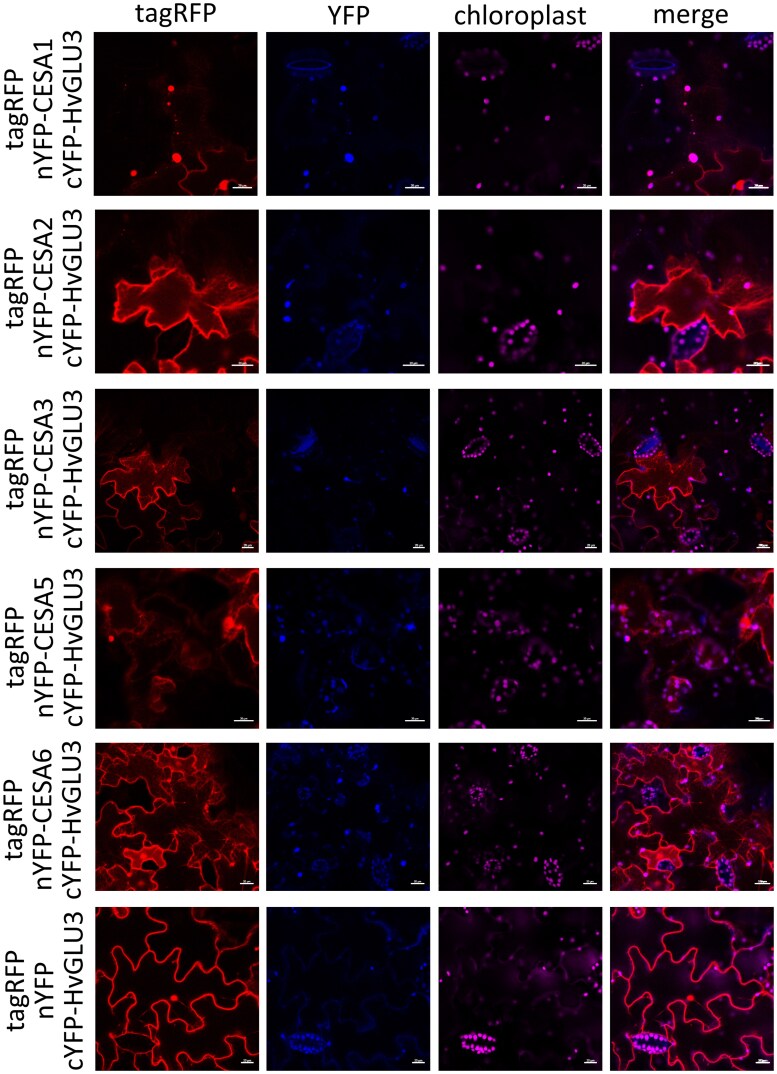
HvGLU3 interacts with primary CESAs. BiFC assay of HvGLU3 and primary cellulose synthases. nYFP, N-terminal part of YFP; cYFP, C-terminal part of YFP. CESA1, HORVU.MOREX.r3.6HG0550800.1; CESA2, HORVU.MOREX.r3.1HG0027850.1; CESA3, HORVU.MOREX.r3.2HG0111610.1; CESA5, HORVU.MOREX.r3.5HG0522880.1; CESA6, HORVU.MOREX.r3.5HG0530450.1. Construct with nYFP and cYFP-HvGLU3 (the last panel) was used as negative control. The tagRFP served as an indicator of successful tobacco infiltration. **A**, **B)** tagRFP, red fluorescence; mVenus/YFP, yellow fluorescence; chloroplasts, autofluorescence of chloroplasts. Scale bar = 20 *µ*m.

To validate the direct interaction between HvGLU3 and the primary CESAs, we performed a BiFC assay. We generated constructs containing the N-terminal part of yellow fluorescent protein (YFP) fused to the N-terminus of the primary CESAs (nYFP-CESA1/2/3/5/6) and the C-terminal part of YFP fused to the N-terminus of HvGLU3 (cYFP-HvGLU3) and infiltrated these constructs into *N. benthamiana* leaves. We observed a YFP signal in *N. benthamiana* epidermal cells transiently cotransformed by HvGLU3 and the different primary CESAs, illustrating direct interactions between HvGLU3 and these CESAs (Fig. 4).

### The defect in cellulose biosynthesis in the mutant *hvglu3-1* leads to a substantial transcriptomic reprogramming across various root tissues

**Figure 5.**
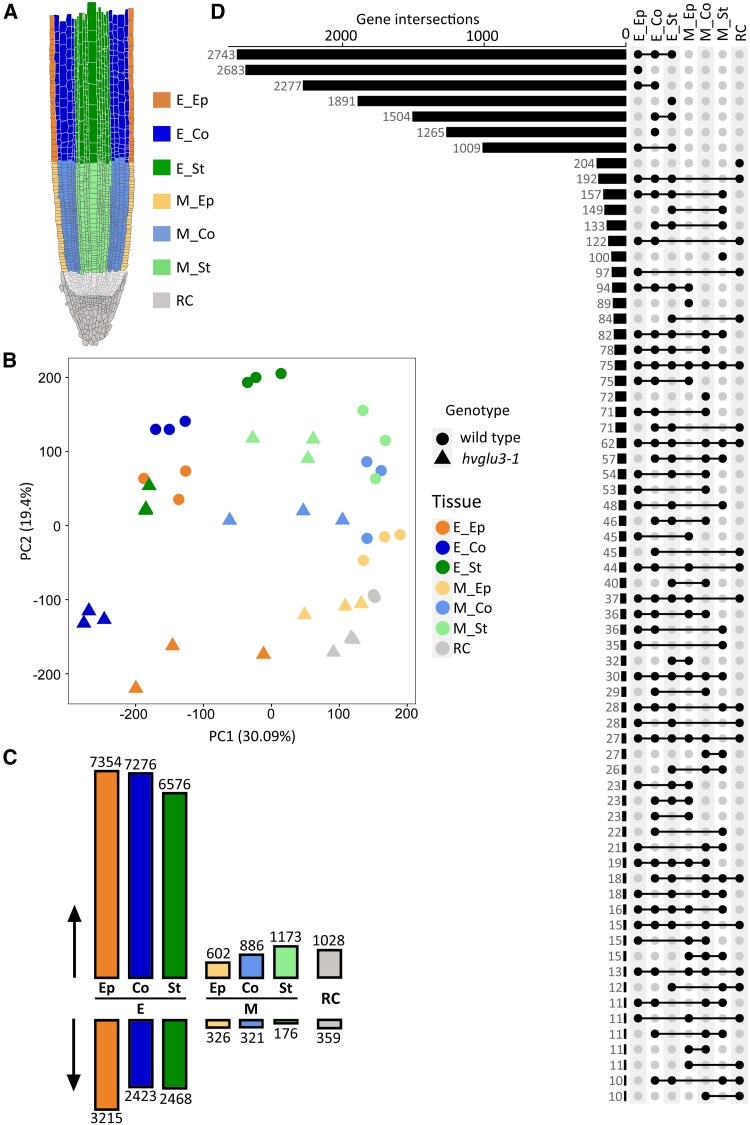
Overview of the RNA-seq. **A)** Experimental setup: the root cap (RC), stele of the meristem (M_St), cortex of the meristem (M_Co), epidermis of the meristem (M_Ep), stele of the elongation zone (E_St), cortex of the elongation zone (E_Co), and epidermis of the elongation zone (E_Ep) were separated using LCM. **B)** PCA plot for the transcriptomes of 3 replicates of the 2 genotypes and 7 root tissues. **C)** Numbers of upregulated (↑) and downregulated (↓) genes determined by pairwise comparisons between the root tissues of the mutant *hvglu3-1* and WT. RC, root cap; M, meristem; E, elongation zone; Ep, epidermis; Co, cortex; St, stele. **D)** Overlap of differentially expressed genes in the 7 tissues determined by pairwise comparisons between the mutant *hvglu3-1* and WT. The size of each intersection is indicated next to the bar.

To explore the impact of defect in cellulose biosynthesis and the resulting changes in cell wall composition on the root transcriptome, we conducted a RNA-seq experiment. First, we isolated the root cap, epidermis, cortex, and stele tissues from the meristem and the elongation zone of seminal roots from WT and the mutant *hvglu3-1* by laser capture microdissection (LCM) (Fig. 5A). After filtering out the lowly expressed genes, which did not have counts per million values of ≥1.1 in at least 3 samples, we analyzed the expression of the remaining 22,389 genes. We examined transcriptomic relationships between genotypes and tissues by a principal component analysis (PCA). In the PCA plot, the 3 biological replicates for each tissue by genotype combination clustered together (Fig. 5B). The first component (PC1), explaining 30% of the total variance, primarily separated samples from different root zones (Fig. 5B).

Subsequently, we determined genes differentially expressed between *hvglu3-1* and WT for each tissue by computing 7 pairwise comparisons. Genes with a log_2_ fold-change (|log_2_FC|) ≥ 1 and a False Discovery Rate of <5% were defined as differentially expressed. In the root cap, 1,387 genes (6% of all detected genes); in the meristem, 2,532 genes (11%); and in the elongation zone, 16,673 genes (74%) exhibited significant differential expression between the mutant *hvglu3-1* and WT (Fig. 5C; Supplementary Table S1). In all 7 tissues, the number of upregulated genes exceeded that of downregulated genes by 2- to 3-fold (Fig. 5C). Through a comparative analysis of differentially expressed gene sets across tissues, we identified genes that exhibited differential expression levels within specific zones or tissues (Fig. 5D).

To investigate the molecular regulation of cell wall composition of the mutant *hvglu3-1* roots, we examined the expression of selected genes involved in cellulose and lignin metabolism in RNA-seq data. Consistent with the results of cell wall component analyses (Fig. 3, F, G, and I), the majority of genes associated with cellulose metabolism were significantly downregulated in the elongation zone of mutant *hvglu3-1* roots (Supplementary Fig. S7A). In contrast, most genes related to lignin metabolism were upregulated in mutant *hvglu3-1* roots (Supplementary Fig. S7B).

### Coexpression analysis determined gene modules strongly associated with tissue types

To identify genes significantly associated with the development of the studied tissue types, we conducted a weighted gene correlation network analysis (WGCNA) and identified 18 modules significantly associated with at least one of the tissue types (Supplementary Fig. S8A and Table S2).

For further analyses, we selected the module with the strongest association with the corresponding tissue type. The expression of the module eigengene is commonly used as a representative of the gene expression pattern within the module. In selected modules, we observed a distinct expression level of eigengene in the corresponding tissue type compared with the other tissue types, except for the 2 modules that were negatively correlated with the cortex of both the meristem and the elongation zone (Supplementary Fig. S8, B to H). Next, we determined the hub genes within each selected module, which exhibit high connections with other genes within a module and closely linked to biological processes, and examined their expression level in the RNA-seq dataset (Supplementary Fig. S8I). Fisher's exact test showed that more differentially expressed genes were enriched in the hub genes of the elongation zone epidermis-associated module Skyblue, the meristem cortex-correlated module Darkmagenta, and the root cap-related module Green than expected (Supplementary Fig. S8I).

### Coexpression analysis determined gene modules strongly associated with genotypes

**Figure 6.**
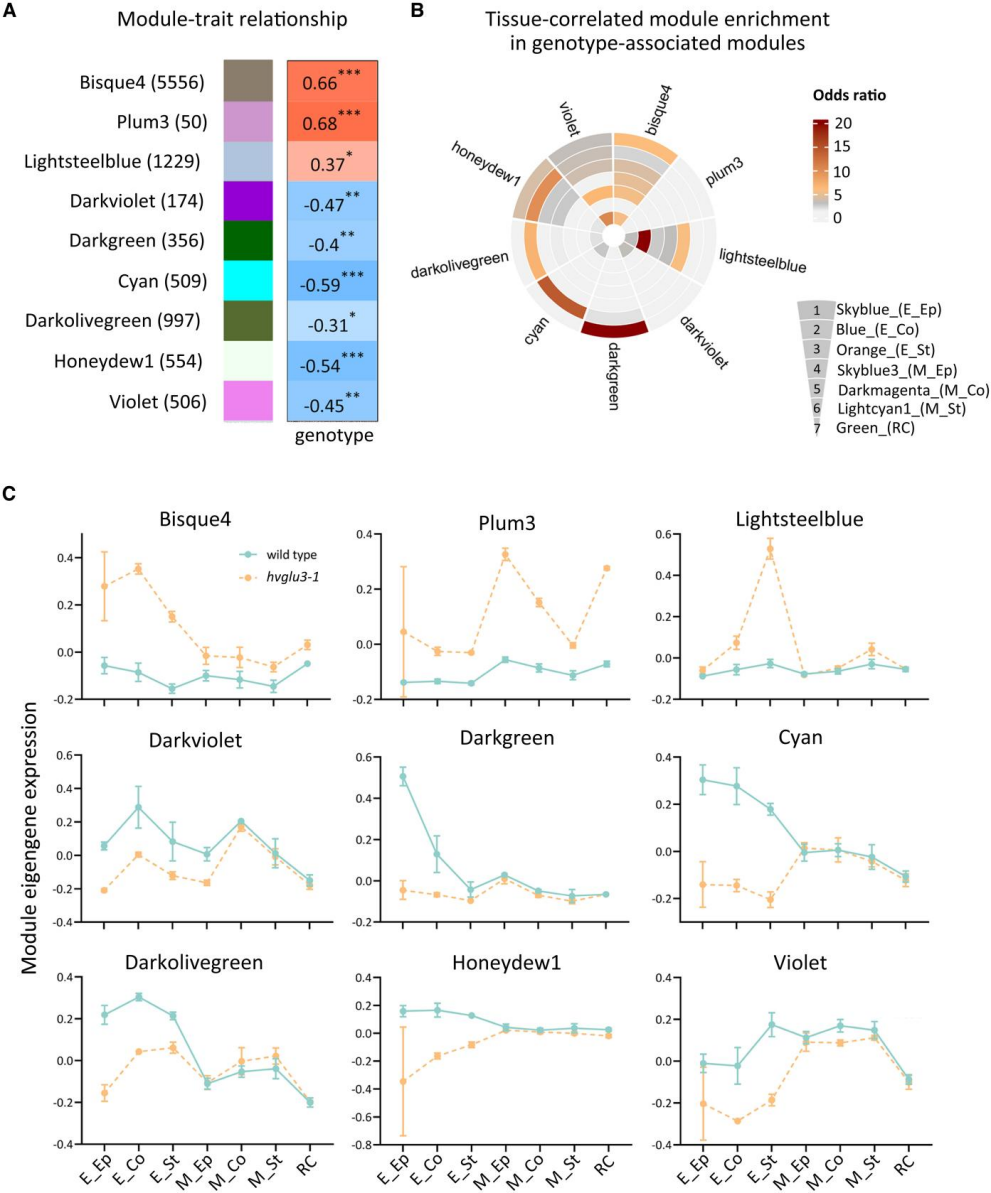
Identification of coexpression networks strongly correlated with genotype and highly associated with tissue development. **A)** Module–trait relationships for genotype, which was designated as trait. Each row represents a coexpression network (module). The relationship between the modules and genotype is calculated by Pearson correlation coefficients and indicated in cells. Asterisks indicate significant values calculated using the corPvalueStudent function. *P < 0.05; **P < 0.01; ***P < 0.001. The number of genes contained in each module is indicated in brackets next to the module names. **B)** Association analysis of modules significantly correlated with genotype and modules strongly associated with tissues. The association between each pair of lists was evaluated using Fisher's exact test and indicated by the odds ratio via cell color. **C)** Eigengene expression of modules that significantly correlated with genotype. *n* = 3 for each genotype. Sd is depicted.

To determine gene networks strongly correlated with genotypes, we performed a WGCNA for all samples, resulting in the identification of 9 modules (Fig. 6A; Supplementary Table S3). Gene Ontology (GO) analyses suggested that genes in these modules are involved in a wide range of biological processes (Supplementary Table S4). To investigate the involvement of these modules in tissue development, we determined the intersections between genotype-associated modules and modules strongly correlated with tissue types (Fig. 6B). The results showed that the 7 genotype-related modules highly overlapped with at least 1 module closely related to the tissue types, suggesting a role for genes in these modules in regulating the development of certain tissues downstream of *HvGLU3* (Fig. 6B). Subsequently, we examined the eigengene expression of genotype-correlated modules (Fig. 6C). The results identified several modules where genes significantly overlap with specific tissue type-associated modules and exhibit distinct expression patterns between genotypes within these tissue types. These modules include Bisque4, Plum3, Lightsteelblue, Darkgreen, and Cyan.

By comparing the top 10 enriched biological process terms for these modules, we observed significant enrichment of genes involved in “hydrogen peroxide catabolic process (GO: 0042744),” “response to oxidative stress (GO: 0006979),” “protein phosphorylation (GO: 0006468),” and “carbohydrate metabolic process (GO: 0005975)” in genotype-related modules whose eigengenes show distinct expression patterns in the tissues of the elongation zone of the 2 genotypes (Supplementary Fig. S9; Fig. 6C). Genes involved in “xyloglucan metabolic process (GO: 0010411)” and “positive regulation of intracellular transport (GO: 0032388)” were enriched in the genotype-associated module Darkgreen, which shows high overlap with the epidermis-specific module Skyblue in the elongation zone (Fig. 6, B and C; Supplementary Fig. S9). Terms related to phytohormone responses were assigned to the module Bisque4, whose eigengenes display distinct expression patterns in the elongation zone of the WT and the *hvglu3-1* mutant (Supplementary Fig. S9; Fig. 6C). The term “lignin catabolic process (GO: 0046274)” was assigned to genes belonging to the module Lightsteelblue, which show preferential expression in the stele of the elongation zone of the *hvglu3-1* mutant (Supplementary Fig. S9; Fig. 6C). Furthermore, several transport-related terms were assigned to the module Cyan, whose genes also display distinct expression patterns in the elongation zone between the 2 genotypes (Supplementary Fig. S9; Fig. 6C). Taken together, these analyses provide a valuable foundation for further studies aimed at gaining a more comprehensive understanding of how defects in cellulose biosynthesis of root cell wall organization impact root tissue development.

## Discussion

Cellulose is the predominant biopolymer and the core structural component within the plant cell wall (Lampugnani et al. 2019; Etale et al. 2023). The biosynthesis of cellulose in plant cell walls by CSCs is controlled by a sophisticated regulatory framework (Li et al. 2014; Polko and Kieber 2019; Pedersen et al. 2023). The identification and characterization of mutants of the *KOR1* gene in *Arabidopsis* (His et al. 2001; Lane et al. 2001; Sato et al. 2001; Paredez et al. 2008; Lei et al. 2014), and its homologs in rice (Zhou et al. 2006; Inukai et al. 2012; Zhang et al. 2012) and sorghum (Mendu et al. 2022), have provided insights into the role of endo-1,4-ß-glucanases in cellulose biosynthesis. However, the mechanism underlying the function of endo-1,4-ß-glucanases remains unclear.

Here, we identified the short root mutant *hvglu3-1* from a chemically mutagenized population of barley (Talamè et al. 2008). As a result of disrupted cell division and inhibited cell elongation, the mutant *hvglu3-1* displays short seminal and crown roots with a rough surface. The epidermis is the primary site for communication with the surrounding environment and for phytohormone action in regulating plant tissue development (Savaldi-Goldstein et al. 2007; Javelle et al. 2011; Short et al. 2018; Vaseva et al. 2018). We observed a thickened epidermis as soon as this cell-type differentiated, followed by epidermal cell loss occurring from the middle of the meristem of mutant *hvglu3-1* roots. This suggests a defect not only in epidermis maintenance but also in its formation. Consequently, the damaged root epidermis is likely the reason for the dramatically restricted root elongation observed in the mutant *hvglu3-1*.

The gene *HvGLU3* encodes a putative membrane-bound endo-1,4-ß-glucanase orthologous to rice OsGLU3. All 3 mutants of *OsGLU3* identified so far displayed defective root elongation while maintaining normal shoot development (Inukai et al. 2012; Zhang et al. 2012). In contrast, the barley mutant *hvglu3-1* displayed a significantly smaller aboveground part and reduced seed production, whereas the *hvglu3-2* mutant exhibited extremely repressed shoot and root development. This indicates a functional diversification of these genes in rice and barley, and that different amino acids contribute variably to the function of these proteins. The normal leaf structure and cellulose accumulation in the shoots suggest that the aboveground phenotype of *hvglu3-1* likely results from restricted root growth, despite *HvGLU3* expression in aboveground tissues. In isoxaben-treated WT roots, we observed an epidermal phenotype similar to that of untreated mutant *hvglu3-1* roots, suggesting that the damaged epidermis in mutant *hvglu3-1* roots was due to a reduction in cellulose content. We observed a loss of ∼60% in crystalline cellulose content in mutant *hvglu3-1* roots compared to WT roots, a more significant reduction than observed in the rice alleles *osglu3-1* and *osglu3-2* (Zhang et al. 2012). In contrast, a slight but significant increase in cellulose content was detected in an additional mutant allele *root growth inhibiting* (*rt*) of the *OsGLU3* gene (Inukai et al. 2012). The contradictory changes in cellulose content observed in different mutant alleles of the same gene are not consistent with the role of *OsGLU3* in cellulose synthesis. The best-studied membrane-bound endo-1,4-ß-glucanase is KOR1 in *Arabidopsis*. The *kor1* mutants display severe phenotypes, including inhibited elongation of hypocotyls and roots along with a reduction in cellulose content compared to the WT (Nicol et al. 1998; Zuo et al. 2000; Lane et al. 2001; Lei et al. 2014). Given the divergent cellulose content changes observed in the rice *rt* mutant and *Arabidopsis kor1* alleles, it was proposed that the *OsGLU3* gene might have a distinct function than *KOR1* (Inukai et al. 2012). It has been suggested that the endoglucanase activity of KOR1 may “edit” the nascent glucan chains of synthesized cellulose microfibrils by relieving tensional stress occurring during the assembly of the microfibrils and/or removing noncrystalline portions of the polymer (Mølhøj et al. 2002). Nevertheless, the mechanism by which endo-1,4-ß-glucanases regulate cellulose biosynthesis remains unexplored. Therefore, the dramatic decrease in cellulose content in *hvglu3-1* mutant roots provided a powerful reference to dissect the role of endo-1,4-ß-glucanases.

The direct interaction between HvGLU3 and primary CESAs suggests that HvGLU3 is involved in cellulose biosynthesis in the root primary cell wall. This is in line with the ubiquitous expression of *HvGLU3* in the root tip, including the root cap, meristem, and elongation zone. Notably, higher expression levels of *HvGLU3* were detected in the elongation zone, likely reflecting the increased cellulose deposition required in this region to provide tensile strength for cell elongation (Tsang et al. 2011). Moreover, the significantly lower expression levels of genes encoding CESAs required for the formation of functional CSCs in the primary cell wall, observed in the elongation zone of the mutant *hvglu3-1*, imply a disruption in CSC assembly and activity. This highlights the critical role of HvGLU3 in these processes. In *Arabidopsis*, KOR1 was proposed to be part of the CSCs via directly interacting with CESAs to regulate cellulose synthesis in both primary and secondary cell walls (Bashline et al. 2014; Lei et al. 2014; Vain et al. 2014). However, Vain et al. (2014) suggested that interactions between CESAs and KOR1 might be transient, given the partial overlap in their localization (Vain et al. 2014). In our study, we observed almost complete colocalization between HvGLU3 and primary CESAs, along with their interaction in punctate structures in the cytoplasm, which are likely part of the *trans*-Golgi network. Notably, interaction signals between HvGLU3 and primary CESAs were not detected at the plasma membrane, despite the membrane localization of HvGLU3 and primary GESAs. In contrast, the AtKOR1–AtCESA6 interaction was observed in similar punctate structures and plasma membrane (Vain et al. 2014). CSCs are assembled in the endoplasmic reticulum and delivered to the plasma membrane via the *trans*-Golgi network (Pedersen et al. 2023). The detection of interaction signals between HvGLU3 and primary CESAs in the *trans*-Golgi network suggests a potential role for HvGLU3 in the trafficking of primary CSCs. However, the absence of detectable interaction signals at the plasma membrane implies that additional mechanisms might be involved in regulating the translocation of CSC complexes to this site. In *Arabidopsis*, KOR1 localization is dynamically controlled by multiple mechanisms during plant growth (Nagashima et al. 2020). Therefore, the heterogeneous interaction pattern observed between HvGLU3 and primary CESAs in *N. benthamiana* might not fully represent their native distribution in barley.

In addition to cellulose, we observed changes in other cell wall polysaccharides in the mutant *hvglu3-1*, including a larger proportion of the hemicellulose arabinoxylan, an altered lignin composition, and a significantly decreased content of aliphatic suberin. In the plant cell wall, hemicelluloses bind to the cellulose microfibrils to form a tight cellulose–hemicellulose network mediating cell elongation and plant growth (Obel et al. 2006; Etale et al. 2023). Lignin is one of the main components of plant secondary cell wall, contributing during cell differentiation through enhancing cell wall strength and rigidity and mediating hydrophobic properties (Liu et al. 2018). Consistent with our results, ectopic lignification triggered by impaired cellulose biosynthesis has been observed in several studies (Caño-Delgado et al. 2003; Rogers et al. 2005). S-rich lignin exhibits lower crosslinking, resulting in a robust and flexible polymer (Bonawitz and Chapple 2010; Verma and Dwivedi 2014). Moreover, S-lignin is preferentially accumulated in infected tissues, suggesting a role in mediating the resistance to pathogen infection (Cesarino 2019). The elevated S-lignin content observed in the mutant *hvglu3-1* is likely a compensatory response to disrupted cell wall organization and impaired epidermal cell wall integrity. Suberin is an intricate polyester present in root epidermal and endodermal cells (Etale et al. 2023). The aliphatic suberin is closely related to water and solute permeabilities (Schreiber et al. 1999; Ranathunge and Schreiber 2011). The substantial changes in the content of these cell wall components in mutant *hvglu3-1* roots suggest alterations in cell wall functionalities, including strength, transport, and permeability. Particularly, the accumulation of rhamnose, a key component of pectins, was significantly higher in the mutant roots compared to the WT, suggesting potential changes in cell wall adhesion. This observation partially aligns with the proposed role of OsGLU3 in facilitating root cap exfoliation from the epidermal cell layer (Inukai et al. 2012). Additionally, similar to the mutation in the mutant *hvglu3-1*, the mutation in *AtCESA6* resulted in reduced cellulose production and an increase in hemicellulose polysaccharides in the *prc1-1* mutant (Fagard et al. 2000). This suggests that the altered contents of other cell wall polysaccharides in mutant *hvglu3-1* roots are likely secondary effects of the deficient cellulose biosynthesis. All changes in these polysaccharides lead to complex cell wall reconstruction, resulting in disrupted epidermal morphology and inhibited root cell division and elongation in the mutant *hvglu3-1*.

The pronounced impact of the mutation in *HvGLU3* on root development is reflected in the enormous remodeling of the root transcriptome in the mutant *hvglu3-1*. Approximately 74% of all active genes were differentially expressed in the elongation zone of mutant *hvglu3-1* seminal roots. However, the substantially transcriptomic reprogramming is likely the consequence of the defects in cellulose biosynthesis rather than being directly regulated by *HvGLU3*. Through GO analyses, we observed hundreds of GO terms were assigned to the differentially expressed genes. Among them, cell wall- and metabolism-related biological process terms were significantly enriched in differentially expressed genes in all 7 tissues of mutant *hvglu3-1* roots compared with WT. Moreover, phytohormone-related terms were also widely enriched in differentially expressed genes. The epidermis is the action site for auxin (Swarup et al. 2005), brassinosteroids (Savaldi-Goldstein et al. 2007; Hacham et al. 2011), and ethylene (Zluhan-Martínez et al. 2021). Biological process terms related to auxin and ethylene responses were significantly enriched in the upregulated genes, while terms related to brassinosteroid response were enriched in downregulated genes in the epidermis of the elongation zone of the mutant *hvglu3-1*. These findings suggest that the primary disturbances in this mutant are associated with cell wall organization and phytohormone-related biological processes, ultimately influencing whole-plant development.

Root development is orchestrated by intricate molecular networks. Gene coexpression networks strongly correlated with tissue type provide insights into the molecular networks governing tissue growth (Langfelder and Horvath 2008). Eigengene expression analyses (Langfelder and Horvath 2007) and the module hub gene determination (Langfelder and Horvath 2007) revealed the key regulatory gene for corresponding tissue development and are promising target for further investigation. For several tissues (the epidermis of the elongation zone, meristem cortex, and root cap), a higher than expected proportion of hub genes vs. all genes was differentially expressed between *hvglu3-1* mutant and WT. This finding highlights the essential role of these hub genes in mediating normal root tissue development, which relies on precisely regulated cellulose biosynthesis. Through the analysis of genotype-specific gene coexpression networks, we identified genes that respond to disturbances in cellulose biosynthesis in the roots. Determining overlapping genes between genotype-associated and tissue development-associated coexpression networks could help isolate genes specifically involved in coordinating cellulose biosynthesis with root tissue development. Here, we identified 9 genotype-related modules, 7 of which displayed a high degree of overlap with tissue type-associated modules. For example, genes in the genotype-associated module Darkgreen displayed distinct overlap with genes in the elongation zone epidermis-related module Skyblue and exhibited high expression levels in the elongation zone epidermis of WT. GO analyses assigned terms related to cell wall organization and oxidative stress/stimulus responses to genes in the module Darkgreen. These findings align with phenotypic observations, uncovering genes involved in linking cellulose biosynthesis or cell wall organization to cellular activity and root development. In this way, these analyses provide promising targets for further investigation into the molecular mechanisms underlying root development, whether dependent on or independent of cellulose biosynthesis.

## Materials and methods

### Plant material and growth conditions

The barley (*H. vulgare* L.) short root mutant *hvglu3-1* (original code TM390) was identified during a paper roll-based screening for root phenotypes of the chemically (sodium azide) mutagenized TILLMore population (Talamè et al. 2008). WT Morex and mutant *hvglu3-1* seeds were incubated on a wet filter paper overnight at 30 °C in darkness and then transferred in paper rolls or 2D rhizoboxes in distilled water in growth cabinets (Conviron, Winnipeg, MB, Canada) at 18 °C during the night (8 h) and 22 °C during the day (16 h). For MutMap+ analysis, WT and *hvglu3-1* plants were grown in the greenhouse, in a peat and vermiculite growing medium (Vigorplant Irish and Baltic peat-based professional mix) in polyethylene pots (15 cm × 15 cm × 20 cm) with the same temperature mentioned above. For isoxaben treatment, seedlings were grown on petri dishes containing ½ strength Hoagland solution supplemented with 0.8% phytagel. Isoxaben (Merck) was dissolved in methanol and added to the ½ strength Hoagland medium to achieve final concentrations mentioned in Results. A medium with the same volume as methanol was used as control. The treated seedlings were grown in growth cabinets for 5 d before measuring their root lengths. To observe the phenotype of adult plants, seedlings were transplanted into soil and grown in a greenhouse until seed harvest.

### Histological analysis

The images of entire roots and shoots were taken with a stereo microscope (Leica, M165 FC). To measure the size of the root cap and the root meristem, 1 cm seminal root tips of 7-d-old (days after germination) WT and the mutant *hvglu3-1* were fixed, stained, and cleared according to the protocol described previously (Truernit et al. 2008). The images of cleared and stained roots were taken with a Zeiss PALM MicroBeam microscope (Zeiss) and analyzed by Fiji (https://imagej.net/software/fiji/). The meristem size was determined by measuring the distance between the root cap and the middle of the transition zone. The transition zone was recognized as described previously (Verbelen et al. 2006).

For leaf sections, the leaf zones were determined and cut as previously described and shown in Results (Li et al. 2010). The 5 mm leaf segments from Zone 3 and Zone 4 of 7-d-old leaves were embedded in 10% (*w*/*v*) agarose. Sections with a thickness of 50 *µ*m were prepared with a vibratome (Leica, VT1200S). For root sections, the 5 mm root segments from the root tip, middle, and base of 7-d-old seminal roots and the 5 mm root segments from the middle of 20-d-old crown roots were embedded and sectioned with a thickness of 100 *µ*m following the same procedure used for leaves. Section images were taken with a Zeiss PALM MicroBeam microscope (Zeiss) and analyzed by Fiji.

Paraffin sectioning and toluidine blue staining were carried out as described in Supplementary Methods S1. Evans blue staining and tetrazolium chloride (TTC) assay were conducted as described in Supplementary Methods S2. Fluorescence staining of sections followed a protocol described previously with minor modification (Ursache et al. 2018; Supplementary Methods S3).

### Mapping *hvglu3-1* with MutMap+

The seeds of the eighth-generation plants produced by 4 seventh-generation plants with intermediate root phenotype were screened with the flat screen method. Seven-day-old seedlings were collected and assigned into mutant (26 plants) or WT (19 plants) bulks based on their root phenotype. The leaf DNA of individual seedling was extracted using the Macherey-Nagel Nucleospin Plant II kit. The same amount of DNA from each WT and mutant seedlings was mixed to obtain 2 bulks with a final concentration of 50 ng/*μ*L. The whole genomic DNA was sequenced with Illumina NovaSeq PE150 (Novogene, Cambridge, UK; Supplementary Methods S4). Raw reads were aligned to the reference Morex v.3 (Mascher et al. 2021) using the bwa mem command of the Burrows-Wheeler Aligner v.0.7.17 (Li and Durbin 2009). Variant calling was done with bcftools v.1.10.2 filtering for a minimum mapping quality of 30 and a minimum base quality of 20 (Danecek et al. 2021). The effect of the called variants was predicted with SNPEff v.5.0c (Cingolani et al. 2012). With a customized shell script, we discarded multiallelic variants and indels and filtered for a minimum coverage of 50× for the mutant bulk and 30× for the WT and minimum PHRED quality of 40 for both. After the variant calling, data were analyzed with the DSNP index method (Takagi et al. 2013), where the index of each SNP is calculated as the ratio between the number of alternate reads at the position and the total number of reads. The DSNP index is the difference between the indexes of the mutant bulk and the index of the WT bulk. We calculated the SNP index for the 2 bulks and their difference and plotted it on the chromosomes using the R ggplot2 package (Wickham 2016).

### FIND-IT screening and isolation of *hvglu3-2*

The FIND-IT cv. RGT Planet library was screened, and a heterozygote targeted barley *hvglu3-2* (HvGLU3^W179Stop^) variant (ID# CB-FINDit-Hv-029) was isolated as described in Knudsen et al. (2022) using an assay with primers listed in Supplementary Table S1. For the detection of the mutant allele, a fluorescently labeled probe 5′6-FAM/CTGAGCTGA/ZEN/TCGGTGG/3′IABkFQ/ was used, while the WT allele was detected with 5′SUN/AGCTGGTCG/ZEN/GTGGT/3′IABkFQ/ (Integrated DNA Technologies, Inc.).

### RT-qPCR

To test the expression of *HvGLU3* (*HORVU.MOREX.r3.2HG0178520.1*) in the root tip, the root zone samples of 3-d-old and 7-d-old seminal roots were separated by a razor blade and frozen immediately in liquid nitrogen. The root segments from 4 roots were collected per plant. Samples from 5 seedlings were pooled as 1 biological replicate. To examine the expression of *HvGLU3* in the entire seedling, 5 mm root segments from the tip, middle, and base, along with the coleoptile, shoot primordia, and 4 leaf zones, were separated and immediately frozen. The leaf zones were determined with modifications as previously described (Li et al. 2010) and categorized as the base zone (Leaf Zone 1, 5 mm above the shoot primordia), the transitional zone (Leaf Zone 2, 5 mm to the first third of the total leaf length), the maturing zone (Leaf Zone 3, 5 mm to 2/3 of the total leaf length), and the mature zone (Leaf Zone 4, 0.5 mm below the leaf tip). Samples from 4 seedlings were pooled as 1 biological replicate. Four biological replicates were used for all tissues. RNA extraction, complementary DNA synthesis, and qPCR preparation were conducted as described previously (Baer et al. 2023). The relative expression levels of *HvGLU3* were calculated with respect to the expression level of the housekeeping gene *TUBULIN* (*HORVU.MOREX.r3.1HG0082050.1*). Oligonucleotide primer sequences were listed in Supplementary Table S5. Significant differences were determined by the Lsd test and labeled with small letters (*P* < 0.05).

### Phylogenetic analysis

The HvGLU3 peptide sequence was blasted against the proteomes of barley, wheat (*Triticum aestivum*), brachypodium (*Brachypodium distachyon*), sorghum (*Sorghum bicolor*), maize (*Zea mays*), rice (*O. sativa*), and *Arabidopsis* (*A. thaliana*) proteome using the Phytozome v13 plant genomics portal (https://phytozome-next.jgi.doe.gov/). Proteins with *E* < 1e^−126^ were selected for phylogenetic tree construction. For the phylogenetic analysis of CESAs in *Arabidopsis* and barley, the peptide sequences of *Arabidopsis* CESA1 to 10 and all proteins annotated as CESA in the *H. vulgare* proteome were used. The peptide sequences of analyzed proteins were downloaded and aligned using the Clustalw function in MEGA11 with default settings (Tamura et al. 2021). The phylogenetic tree for HvGLU3 and its homologs was constructed using the neighbor-joining method with default values via the phylogeny function (Saitou and Nei 1987). For the phylogenetic tree of CESAs, the maximum likelihood method was employed. Only the subtree containing barley proteins that were closely related to *Arabidopsis* CESAs was displayed. Haplotype and association analyses at *HvGLU3* were performed as described in Supplementary Methods S5.

### Plant cell wall analysis

The cellulose content and matrix polysaccharide monosaccharide composition of 7-d-old shoot and root tissues were determined as described (Yeats et al. 2016). Tissues from 5 seedlings were collected and pooled as 1 replicate, and 5 replicates were analyzed per genotype. In brief, alcohol insoluble material (AIR) was prepared and split into 2 samples. One sample was treated with a weak acid (4% sulfuric acid) to release matrix polysaccharide-derived sugars, while the other sample was treated first with a strong acid (72% sulfuric acid) to swell cellulose. Then, the sulfuric acid concentration was diluted to 4% to yield monosaccharides both derived from cellulose and the matrix polymers. Subtraction of the 2 values allows for the quantification of crystalline cellulose. Monosaccharides of all fractions were quantified using a Professional IC Vario high-performance anion-exchange chromatography system 1068 (Metrohm, Herisau, Switzerland) equipped with a CarboPac PA20 column (Thermo Fisher Scientific, Waltham, MA, USA) and an amperometric detector (Metrohm). The used sodium hydroxide gradient is specified as described (Yeats et al. 2016). Lignin and suberin content analyses were performed according to Supplementary Methods S6.

### BiFC assay

For the BiFC assay, the entry vectors (containing attL sites) used for the subcellular localization experiments (described in Supplementary Methods S7) were integrated into the destination vectors pBiFCt-2in1-NN (containing attR site) by LR Clonase reactions. Finally, we generated binary expression constructs containing *HvGLU3* coding sequence C-terminally fused to the C-terminal part of YFP (cYFP-HvGLU3) and the coding sequence of *HvCESAs* C-terminally fused to the N-terminal part of YFP (nYFP-CESAs). Additionally, we generated a construct containing only *HvGLU3* coding sequence C-terminally fused to the C-terminal part of YFP; no sequence fused to the N-terminal part of YFP serving as a negative control. The 35s promoter-driven tagRFP (tagRFP) was included in all vectors as a positive control for successful infiltration into *N. benthamiana*. All verified constructs were transformed into *Agrobacterium* strain AGL1. *N. benthamiana* infiltration and fluorescence analyses were conducted according to the methods described previously (Guo et al. 2023).

### Tissue separation by LCM and RNA isolation for RNA-seq

LCM was employed to collect root tissues, including epidermis, cortex, and stele from the elongation zone and the meristem, as well as the root cap, for RNA-seq. This procedure was conducted following the methods described in a previous study (Kirschner et al. 2021; Supplementary Methods S8).

### RNA-seq and data analysis

RNA amplification was conducted prior to library preparation using the SMARTer kit (Takara Bio) due to the limited RNA quantity. This amplification process was performed following the manufacturer's protocol by Novogene (Novogene, Cambridge, UK). Only samples that passed the amplification assessment and were classified as “normal” or “slightly degraded” were further processed for library construction and sequencing. The library preparation and sequencing were conducted as described previously (Guo et al. 2023). Approximately 9 Gb of raw data was generated for each sample.

The raw RNA-seq data analysis using CLC GENOMICS WORKBENCH (v.23.0.1) and generated data processing were carried out following the methods described previously (Guo et al. 2023; Supplementary Methods S9).

### WGCNA

For determining the tissue type-associated modules, the gene count matrix was sorted from the count data of WT samples generated by CLC GENOMICS WORKBENCH. To identify modules correlated with genotypes, the gene count matrix that included data from both WT and mutant *hvglu3-1* samples was analyzed. Coexpression networks were constructed according to the method mentioned in a previous study (Guo et al. 2023; Supplementary Methods S10).

### Accession numbers

Sequence data from this article can be found in the GenBank/EMBL data libraries under the following accession numbers: HvGLU3 (HORVU.MOREX.r3.2HG0178520.1), CESA1 (HORVU.MOREX.r3.6HG0550800.1), CESA2 (HORVU.MOREX.r3.1HG0027850.1), CESA3 (HORVU.MOREX.r3.2HG0111610.1), CESA4 (HORVU.MOREX.r3.1HG0041280.1), CESA5 (HORVU.MOREX.r3.5HG0522880.1), CESA6 (HORVU.MOREX.r3.5HG0530450.1), and CESA7 (HORVU.MOREX.r3.5HG0482670.1).

## Supplementary Material

kiaf311_Supplementary_Data

## Data Availability

RNA-seq data have been deposited in the SRA under accession number PRJNA1120842 (https://www.ncbi.nlm.nih.gov/bioproject/1120842). Isolated barley FIND-IT variant seeds (*hvglu3-2*, ID# CB-FINDit-Hv-029) can be provided by the Carlsberg Research Laboratory (contact Christoph.Dockter@carlsberg.com) pending scientific review and a completed material transfer agreement.

## Supplementary data

The following materials are available in the online version of this article.

**Supplementary Figure S1.** Metabolic assessment and histological staining of WT and *hvglu3-1*.

**Supplementary Figure S2.** Seminal root phenotype of WT and *hvglu3-1*.

**Supplementary Figure S3.** The phenotype of crown roots and adult plants of WT and *hvglu3-1*.

**Supplementary Figure S4.** Protein sequence alignment of HvGLU3 and its homologs and haplotype and association analysis at *HvGLU3*.

**Supplementary Figure S5.** Levels of lignin monomers and suberin in roots and the negative control used for the histological staining of WT and *hvglu3-1* leaf sections.

**Supplementary Figure S6.** HvGLU3 colocalizes with barley primary CESAs.

**Supplementary Figure S7.** The expression levels of selected genes determined by RNA-seq.

**Supplementary Figure S8.** Coexpression analyses identify gene networks strongly correlated with different tissues.

**Supplementary Figure S9.** GO terms enriched in selected genotype-associated modules

**Supplementary Table S1.** Overview of the genes differentially expressed in the root tissues of *hvglu3-1* compared to WT.

**Supplementary Table S2.** Gene list of modules significantly associated with tissue type.

**Supplementary Table S3.** Gene list of modules significantly associated with genotype.

**Supplementary Table S4.** GO analysis of modules strongly associated with genotype and overlapping with tissue type-related modules.

**Supplementary Table S5.** List of oligonucleotide primers.

**Supplementary Methods S1.** Paraffin sectioning and toluidine blue staining.

**Supplementary Methods S2.** Evans blue staining and TTC assay.

**Supplementary Methods S3.** Histological staining of sections with Direct Red 23, calcofluor white, and basic fuchsin.

**Supplementary Methods S4.** Whole genome sequencing.

**Supplementary Methods S5.** Haplotype and association analysis at *HvGLU3*.

**Supplementary Methods S6.** Quantification of lignin and suberin content.

**Supplementary Methods S7.** Subcellular localization.

**Supplementary Methods S8.** Tissue separation by LCM and RNA isolation for RNA-seq.

**Supplementary Methods S9.** RNA-seq data analysis.

**Supplementary Methods S10.** Weighted gene correlation network analysis (WGCNA).
