# Supplementary material for: The putative endo-1,4-β-D-glucanase GLU3 regulates cellulose biosynthesis in barley roots
Source: Plant Physiol. 2025 Jul 17;198(3):kiaf311. doi: 10.1093/plphys/kiaf311 (PMC12311300; doi:10.1093/plphys/kiaf311)
Supplement: kiaf311_Supplementary_Data [file kiaf311_supplementary_data.zip › PLPHYS-2025-0279R1_Supplementary Data.pdf]

Supplementary Data

Article title: ***HvGLU3* encodes a putative endo-1,4-β-D-glucanase required for cellulose biosynthesis in barley root**

Authors: Li Guo[a], Serena Rosignoli[b], Magnus Wohlfahrt Rasmussen[c], Kiran Suresh[d], Giuseppe Sangiorgi[b], Francesco Camerlengo[b], Viktoria V. Zeisler-Diehl[d], Lukas Schreiber[d], Christoph Dockter[c], Markus Pauly[e], Roberto Tuberosa[b], Frank Hochholdinger[a,*] and Silvio Salvi[b,*].

The following Supplementary Data is available for this article:

Supplementary Figure S1 Metabolic assessment and histological staining of WT (wild type) and *hvglu3-1*.

Supplementary Figure S2 Seminal root phenotype of WT and *hvglu3-1*.

Supplementary Figure S3 The phenotype of crown roots and adult plants of WT and *hvglu3-1*.

Supplementary Figure S4 Protein sequence alignment of HvGLU3 and its homologs and haplotype and association analysis at *HvGLU3*.

Supplementary Figure S5 Levels of lignin monomers and suberin in roots and the negative control used for the histological staining of WT and *hvglu3-1* leaf sections.

Supplementary Figure S6 HvGLU3 colocalizes with barley primary cellulose synthases.

Supplementary Figure S7 The expression levels of selected genes determined by RNA sequencing.

Supplementary Figure S8 Co-expression analyses identify gene networks strongly correlated with different tissues.

Supplementary Figure S9 Gene Ontology (GO) terms enriched in selected genotype-associated modules.

Supplementary Table S1 Overview of the genes differentially expressed in the root tissues of *hvglu3-1* compared to wild type. [submitted separately]

Supplementary Table S2 Gene list of modules significantly associated with tissue type. [submitted separately]

Supplementary Table S3 Gene list of modules significantly associated with genotype [submitted separately].

Supplementary Table S4 Gene Ontology analysis of modules strongly associated with genotype and overlapping with tissue type-related modules [submitted separately].

Supplementary Table S5 List of oligonucleotide primers [submitted separately].

Supplementary Methods S1 Paraffin sectioning and Toluidine blue staining.

Supplementary Methods S2 Evans blue staining and tetrazolium chloride (TTC) assay.

Supplementary Methods S3 Histological staining of sections with Direct Red 23, Calcofluor White and Basic Fuchsin.

Supplementary Methods S4 Whole Genome Sequencing (WGS).

Supplementary Methods S5 Haplotype and association analysis at *HvGLU3*.

Supplementary Methods S6 Quantification of lignin and suberin content.

Supplementary Methods S7 Subcellular localization.

Supplementary Methods S8 Tissue separation by laser capture microdissection and RNA isolation for RNA sequencing.

Supplementary Methods S9 RNA sequencing data analysis.

Supplementary Methods S10 Weighted gene correlation network analysis (WGCNA).

Supplementary References

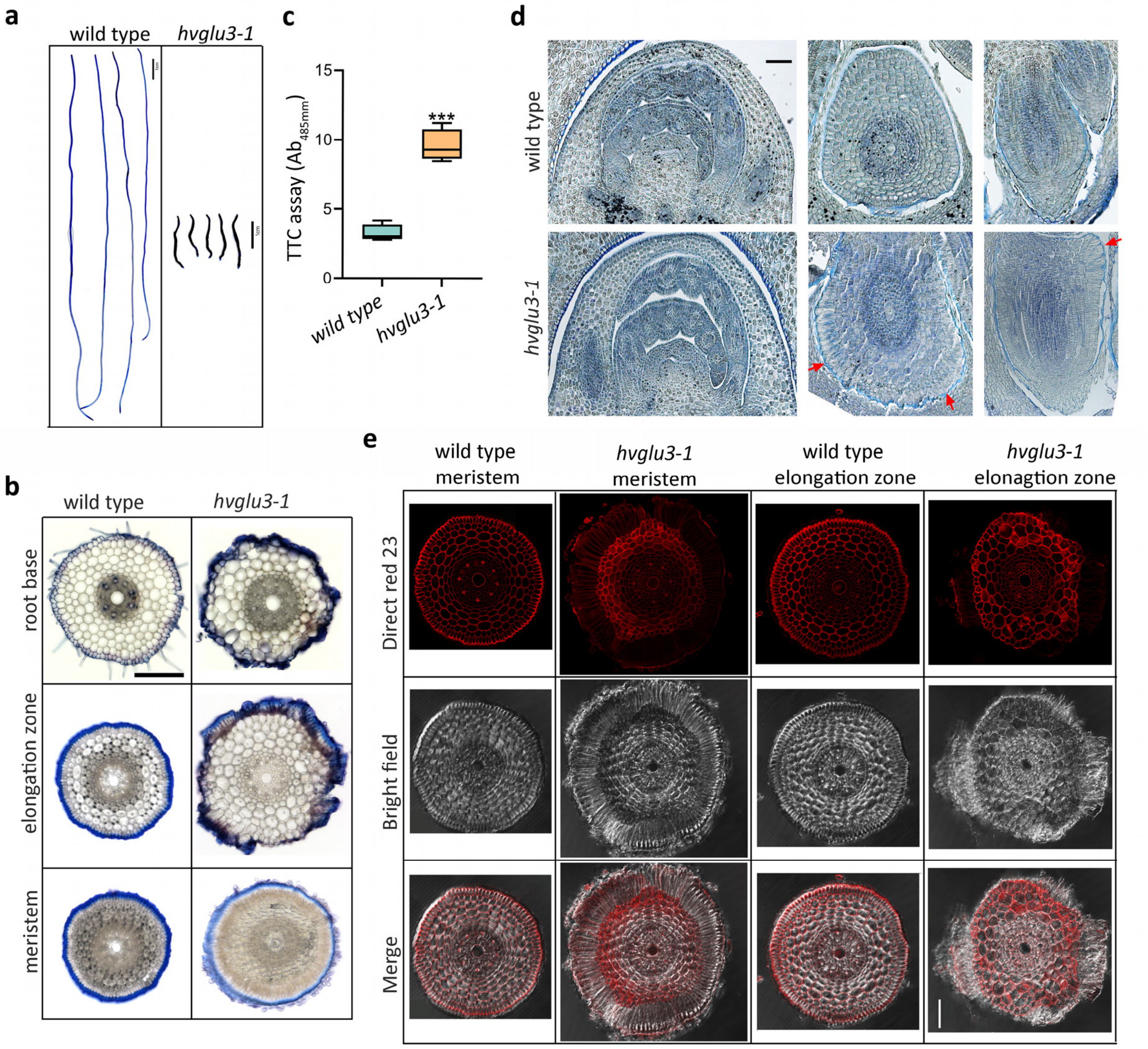

Supplementary Figure S1 Metabolic assessment and histological staining of WT and *hvglu3-1*. **(a)** Detection of cell death in the seminal roots of wild type and the mutant *hvglu3-1* by Evan's blue staining. Scale bar = 1 cm. **(b)** Cross section of the seminal roots stained in (a). Scale bar for all images in (b) = 200 μm. **(c)** Cell metabolic activity test for the seminal roots of wild type and the mutant *hvglu3-1* by triphenyltetrazolium chloride (TTC) assay. Five roots from different seedlings were collected as one biological replicate, three biological replicates were used per genotype per experiment. Two individual experiments were conducted. In the boxplots, the center line within each box represents the median; box limits indicate the upper and lower quartiles; the whiskers extend to the minimum and maximum values. Two-tailed *t* test, ***: $p < 0.001$. **(d)** Toluidine blue staining of paraffin sections from WT and *hvglu3-1* embryos. Red arrows indicate

the thicker epidermis of the mutant *hvglu3-1*. Scale bar for all images in (d) = 100 µm. **(e)** Direct red 23 staining for the cross section of the meristem and the elongation zone of WT and *hvglu3-1* seminal roots. Scale bar for all image in (e) = 100 µm.

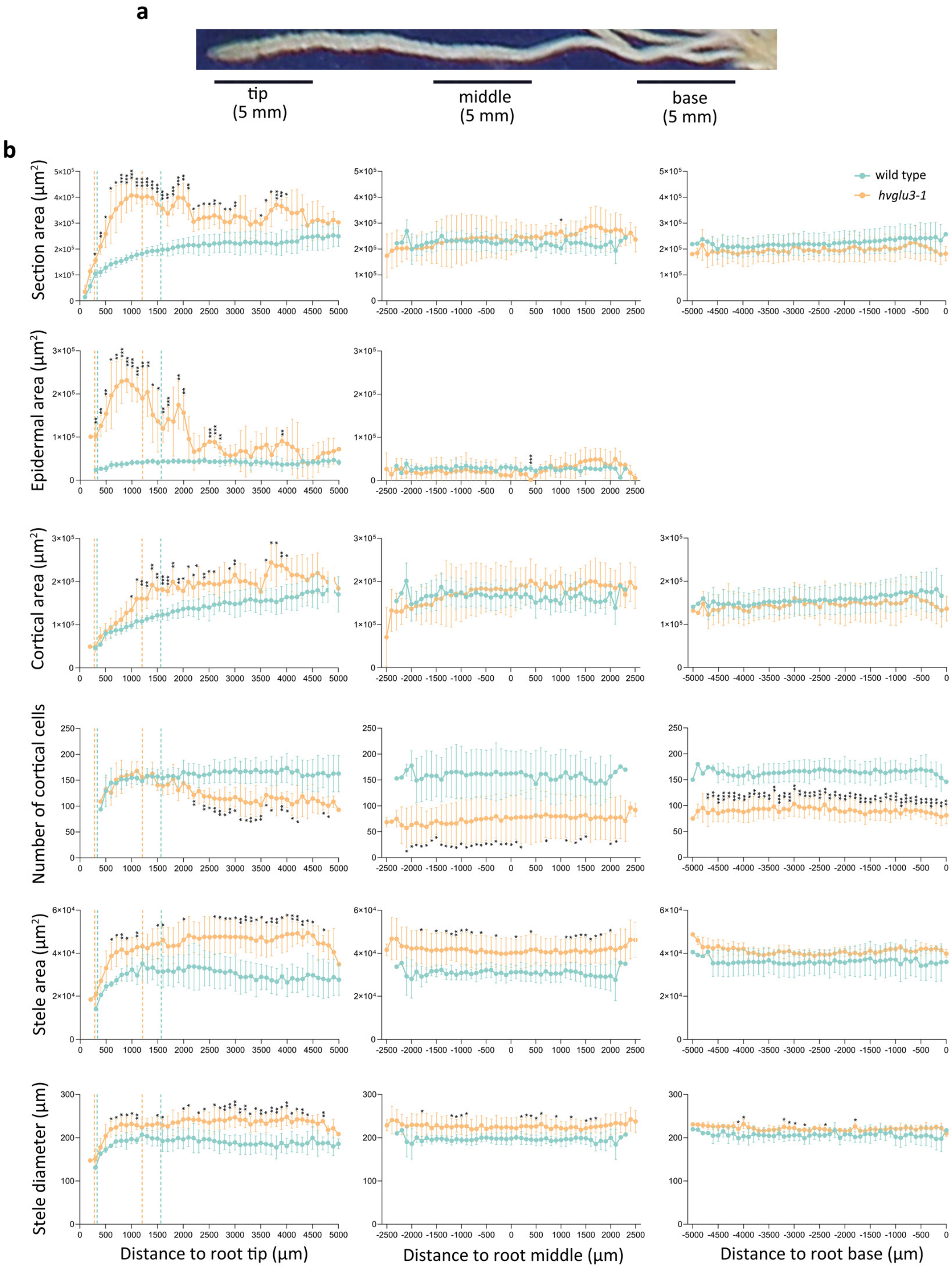
a
tip
(5 mm)
middle
(5 mm)
base
(5 mm)
b
wild type
hvglu3-1
Section area (µm²)
Epidermal area (µm²)
Cortical area (µm²)
Number of cortical cells
Stele area (µm²)
Stele diameter (µm)
Distance to root tip (µm)
Distance to root middle (µm)
Distance to root base (µm)

Supplementary Figure S2 Seminal root phenotype of WT and the mutant *hvglu3-1*. **(a)** Examples of root segments for which different parameters have been determined in (b). **(b)** The section area, epidermal area, cortical area, number of cortical cells, stele area, and the stele diameter in the 0.5 mm root segments from the root tip, the middle of roots, and the basal part of roots were measured. The measurements of sections with the same relative position of the roots were compared between the wild type and the mutant *hvglu3-1* by a two-tailed t test. *: $p < 0.05$; **: $p < 0.01$, ***: $p < 0.001$. n = 4 per genotype. SD is depicted.

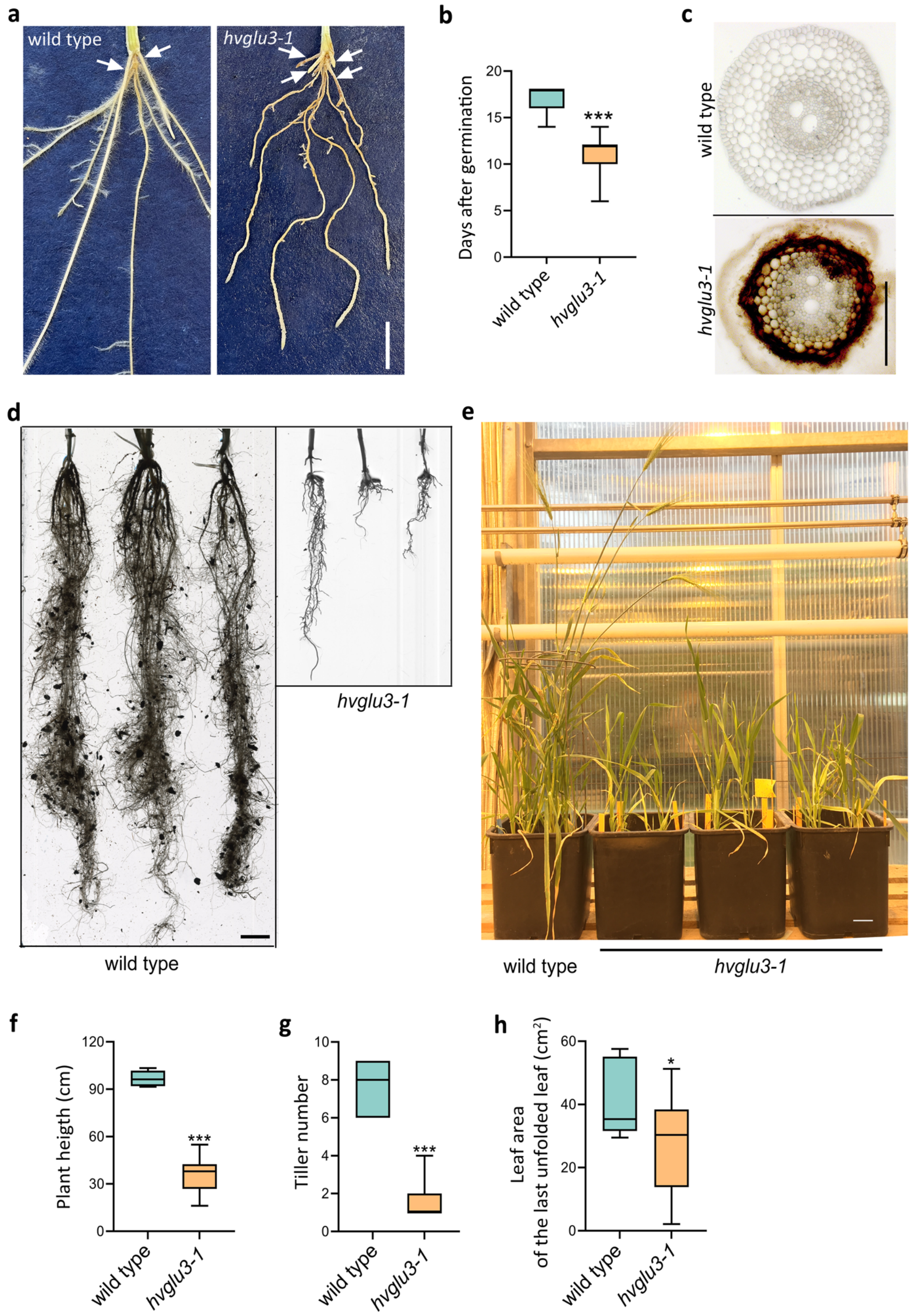

Supplementary Figure S3 The phenotype of crown roots and adult plants of WT and the mutant *hvglu3-1*. **(a)** Root system of 20-day-old seedlings of wild type and the mutant *hvglu3-1*. Arrows indicate the crown roots. Scale bar for both images = 1 cm. **(b)** Time of crown root emergence. n = 8 for WT, n = 5 for the *hvglu3*-1. In the boxplots, the center line within each box represents the median; box limits indicate the upper and lower quartiles; the whiskers extend to the minimum and maximum values. Two-tailed t test, ***: $p < 0.001$. **(c)** Cross sections of wild type and *hvglu3*-

1 mutant crown roots, Scale bar for both images = 300 µm. **(d)** Root system morphology of 60-day-old wild type and the mutant *hvglu3-1* plants. Scale bar for both images = 2 cm. **(e)** Above-ground phenotype of 60-day-old wild type and mutant *hvglu3-1* plants. Scale bar = 5 cm. **(f)** Plant height, **(g)** tiller number, **(h)** and leaf area of the last unfolded leaf of 60-day-old wild type and mutant plants. (f-h) n = 7 for each genotype. In the boxplots, the center line within each box represents the median; box limits indicate the upper and lower quartiles; the whiskers extend to the minimum and maximum values. Two-tailed t test, *: $p < 0.05$; ***: $p < 0.001$.

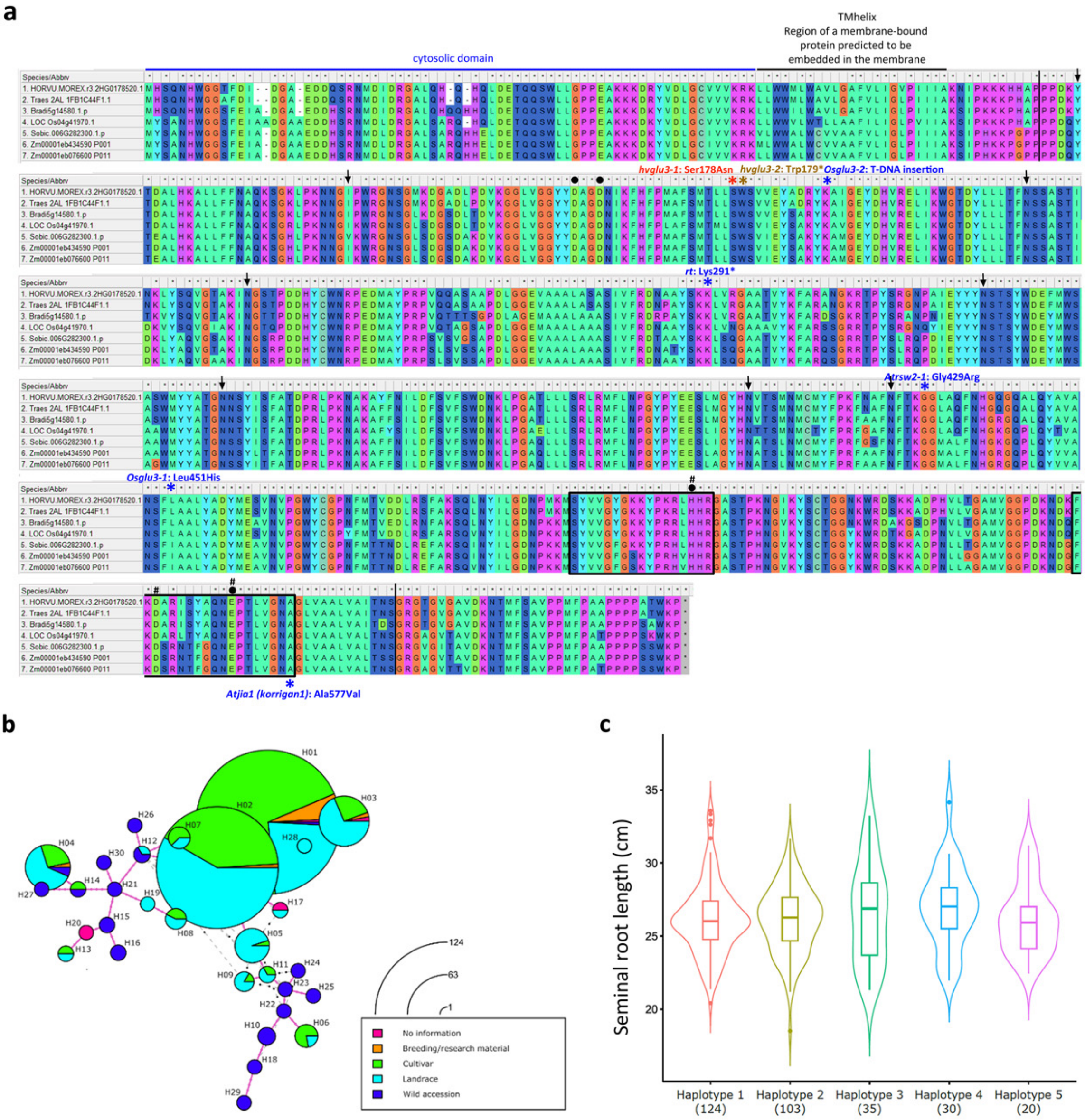

Supplementary Figure S4 Protein sequence alignment of HvGLU3 and its homologs and haplotype and association analysis at *HvGLU3*. **(a)** Alignment of homologs of barley (*Hordeum vulgare*) HvGLU3, wheat (*Triticum aestivum*), brachypodium (*Brachypodium pinnatum*), rice (*Oryza sativa*), sorghum (*Sorghum bicolor*) and maize (*Zea mays*) with a sequence identity > 80%. The cytosolic domain of these proteins is indicated by a blue line. The TMhelix region, the region of a membrane-bound protein predicted to be embeded in the membrane, is indicated by black line. The glycosylation sites of these proteins are depicted by arrows (↓). The catalytic activity sites are depicted by a hashtag (#). Black dots (●) indicate four amino acid residues essential for catalytic activity identified in the *C. thermocellum* endoglucanase CelD, a representative member of the family of E cellulases (Chauvaux et al., 1992). These four amino acid residues are also conserved in proteins listed in phylogenetic the tree shown in Figure 3a. The sequence between two vertical bars (| |) includes six-hairpin glycosidases. Sequences in black boxes are glycosyl hydrolase family 9 active site signature motif 1 and signature motif 2. The asterisks indicate the mutation sites in the mutant *hvglu3-1* and the previously published mutants of HvGLU3 homologs. **(b)** Representation of the 30 *HvGLU3* haplotypes which were identified in the WHEALBI barley germplasm collection. Haplotypes exhibiting high similarity are positioned close to each other; dots along the red lines connecting distinct haplotypes indicate the number of single nucleotide polymorphisms (SNPs) that distinguish them. The size of each pie is proportional to the number of accessions. **(c)** Seminal root length for barley accessions with the five most frequent haplotypes (> 5% frequency). n =10 for each accession. No significant differences were identified among haplotypes for average seminal root length. ANOVA was used as the statistical test, with $p < 0.05$ considered significant. In the boxplots, the center line within each box represents the median; box limits indicate the upper and lower quartiles; the whiskers extend to the minimum and maximum values, Points represent the outliers.

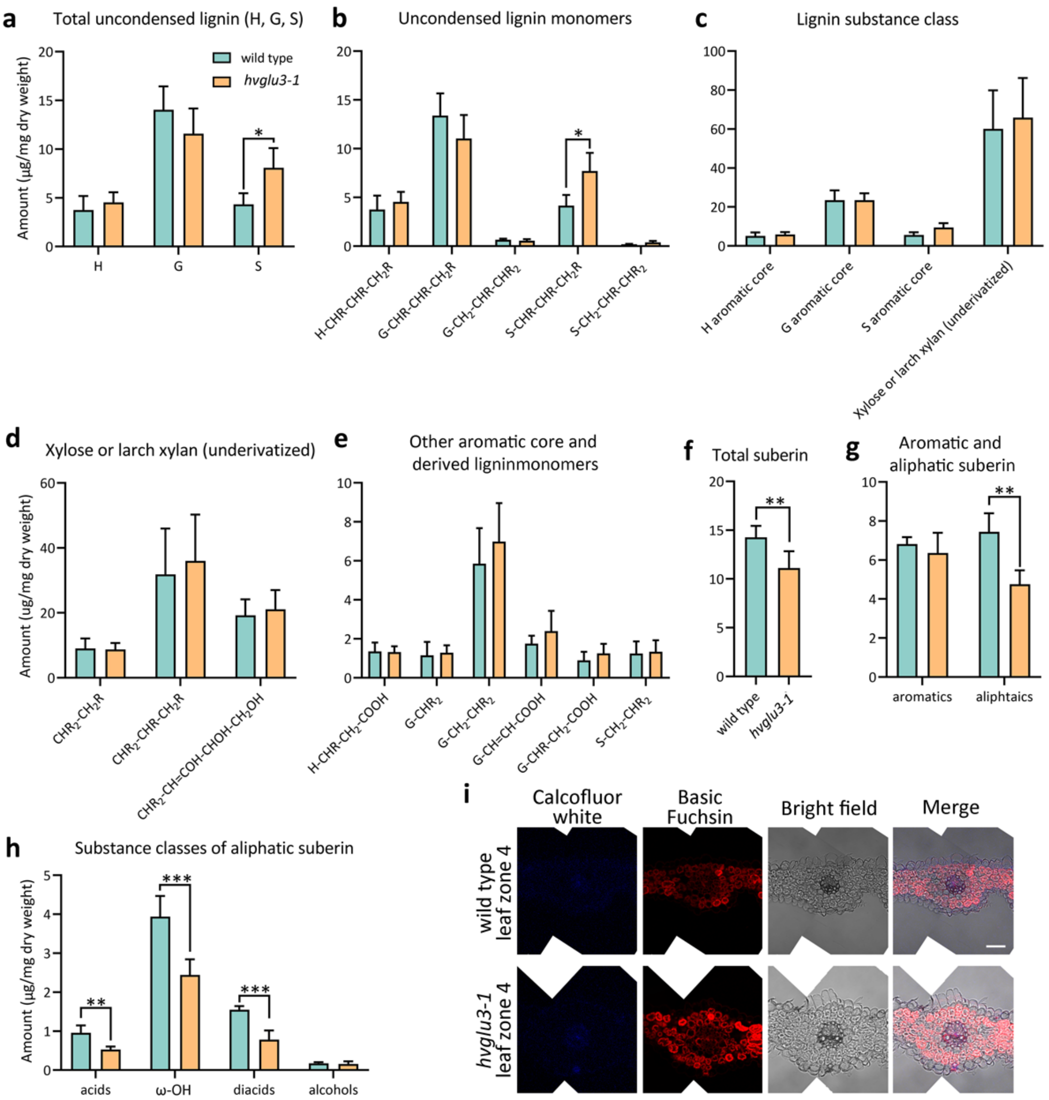

Supplementary Figure S5 Amount of lignin monomers and suberin in seminal roots and negative control of histological staining of leaf section of wild type and the mutant *hvglu3-1*. **(a)** Total uncondensed lignin (H, G, S). H: p-Hydroxyphenyl (4-hydroxyphenyl); G: Guaiacyl (4-hydroxy-3-methoxyphenyl); S: Syringyl (4hydroxy-3,5-dimethoxyphenyl). **(b)** Uncondensed lignin monomers. **(c)** Lignin substance classes. **(d)** Xylose or larch xylan (underivatized) during thioacidolysis process. **(e)** Other aromatic core and derived lignin monomers. **(f)** Total suberin. **(g)** Aromatic and aliphatic suberin. **(h)** Substance classes of aliphatic suberin: fatty acids, ω-hydroxyacids, diacids and alcohols. (a-h) For wild type seedlings, all seminal roots of 9-10 seedlings were collected as one replicate. For mutant *hvglu3-1* seedlings, all seminal roots of 6-8 seedlings were collected as one replicate. Five replicates were analyzed per genotype. SD is

depicted. Two-tailed *t* test, *: $p < 0.05$, **: $p < 0.01$, ***: $p < 0.001$. **(i)** Negative control of Calcofluor white and Basic Fuchsin staining of leaf section of wild type and the mutant *hvglu3-1*. The images are composite images. Scale bar for all images in this panel = 50 µm.

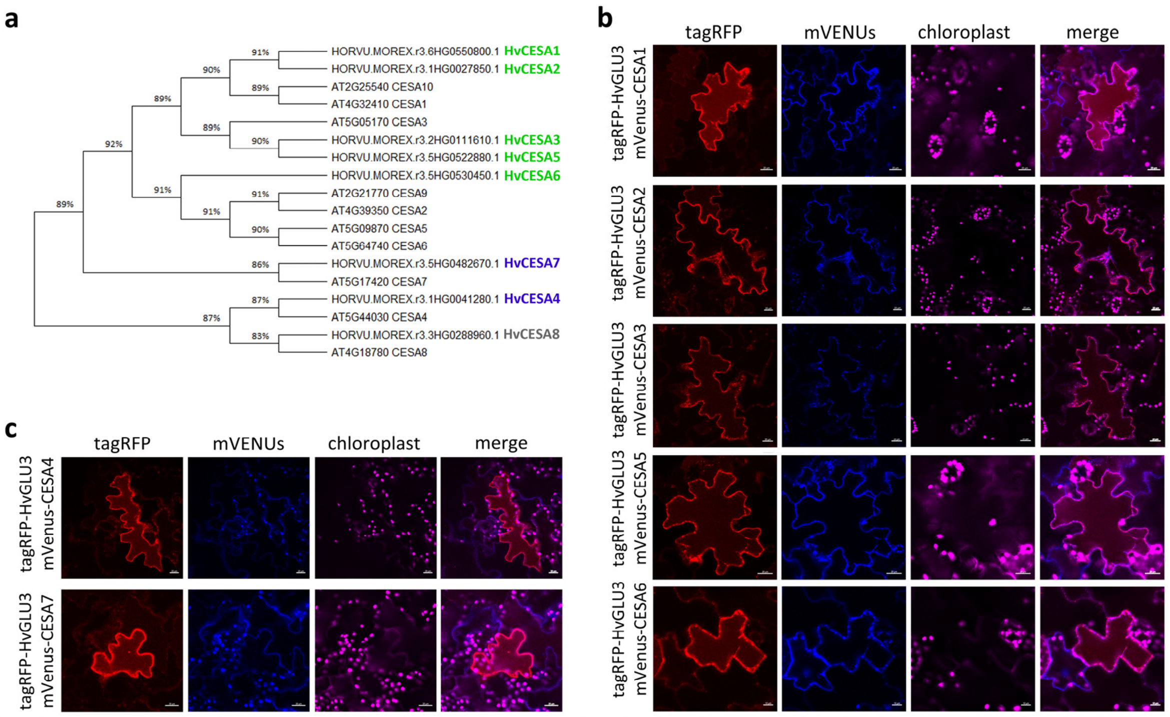

Supplementary Figure S6 HvGLU3 colocalizes with barley primary cellulose synthases. **(a)** Phylogenetic tree for *Arabidopsis* cellulose synthases and their homologous proteins in barley. Primary cellulose synthases in barley are highlighted in green, secondary cellulose synthases in barley are highlighted in blue and grey. Scale bars for all images in this panel = 20 µm. **(b)** Co-localization assay of HvGLU3 with primary cellulose synthases. **(c)** Co-localization assay of HvGLU3 and two of secondary cellulose synthases (highlighted in blue). Scale bars for images in the first four rows = 20 µm. Scale bars for images in the last row = 10 µm. (b-c) tagRFP: red fluorescence, mVenus: yellow fluorescence, Chloroplasts: autofluorescence of chloroplasts.

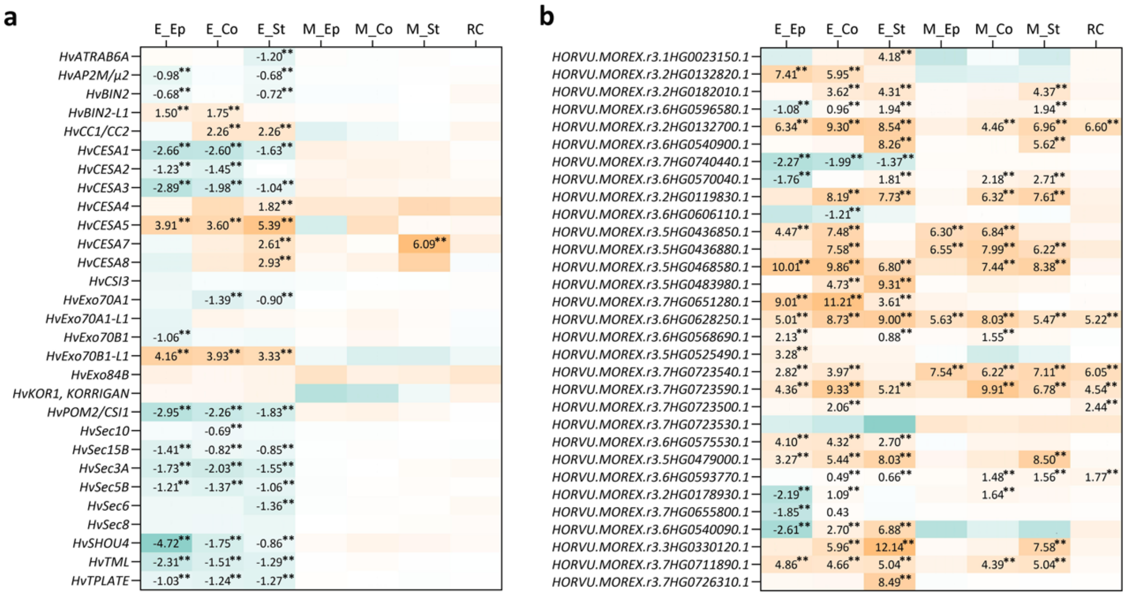

Supplementary Figure S7 The expression levels of selected genes in RNA sequencing data. (a-b) Relative expression of genes involved in cellulose (a) and lignin (b) biosynthesis in the mutant *hvglu3-1* compared with that of the wild type. Upregulation is indicated by orange, and downregulation is indicated by cyan. Log2 fold-change value ($\log_2$FC) is presented in cell, asterisks indicate adjusted *p* value (FDR), **: false discovery rate (FDR) < 5%.

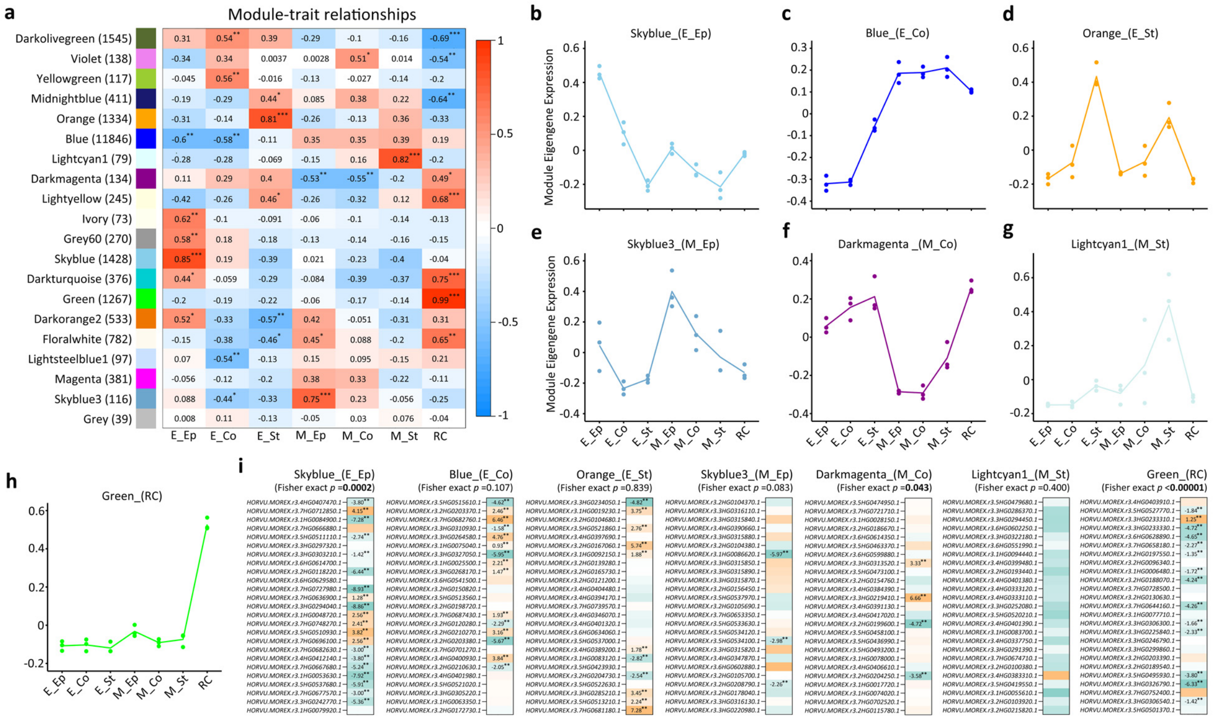

Supplementary Figure S8 Co-expression analyses to identify gene networks strongly correlated with tissues. **(a)** Module-trait relationships for wild type tissues. Each column corresponds to one of the seven tissues, which were designed as trait. Each row corresponds to a co-expression network, known as a module. The relationship between the modules and traits is indicated in cell by Pearson correlation coefficients. Asterisks indicate significant values calculated using the corPvalueStudent function, *: $p < 0.05$; **: $p < 0.01$, ***: $p < 0.001$. Cell color ranges from red (highly positive correlation) to blue (highly negative correlation). The number of genes contained in each module is indicated in brackets next to the module names. **(b-h)** Eigengene expression of modules that are most strongly associated with one of the seven tissues. These selected modules include module Skyblue for epidermis of the elongation zone (E_Ep), module Blue for cortex of the elongation zone (E_Co), module Orange for stele of the elongation zone (E_St), module Skyblue3 for epidermis of the meristem (M_Ep), module Darkmagenta for cortex of the meristem (M_Co), module Lightcyan1 for stele of the meristem (M_St), and module Green for the root cap (RC). **(i)** The expression of hub genes within selected modules in corresponding tissue of *hvglu3-1* compared with that of the wild type. Cell color ranges from orange (upregulation) to cyan (downregulation). The $\log_2$ fold-changes ($\log_2$FC) of hub genes are indicated in cells. **: FDR < 5%. (a-i) E_Ep: epidermis of the elongation zone; E_Co: cortex of the elongation zone; E_St: stele of the elongation zone; M_Ep: epidermis of the meristem; M_Co: cortex of the meristem; M_St: stele of the meristem; RC: the root cap.

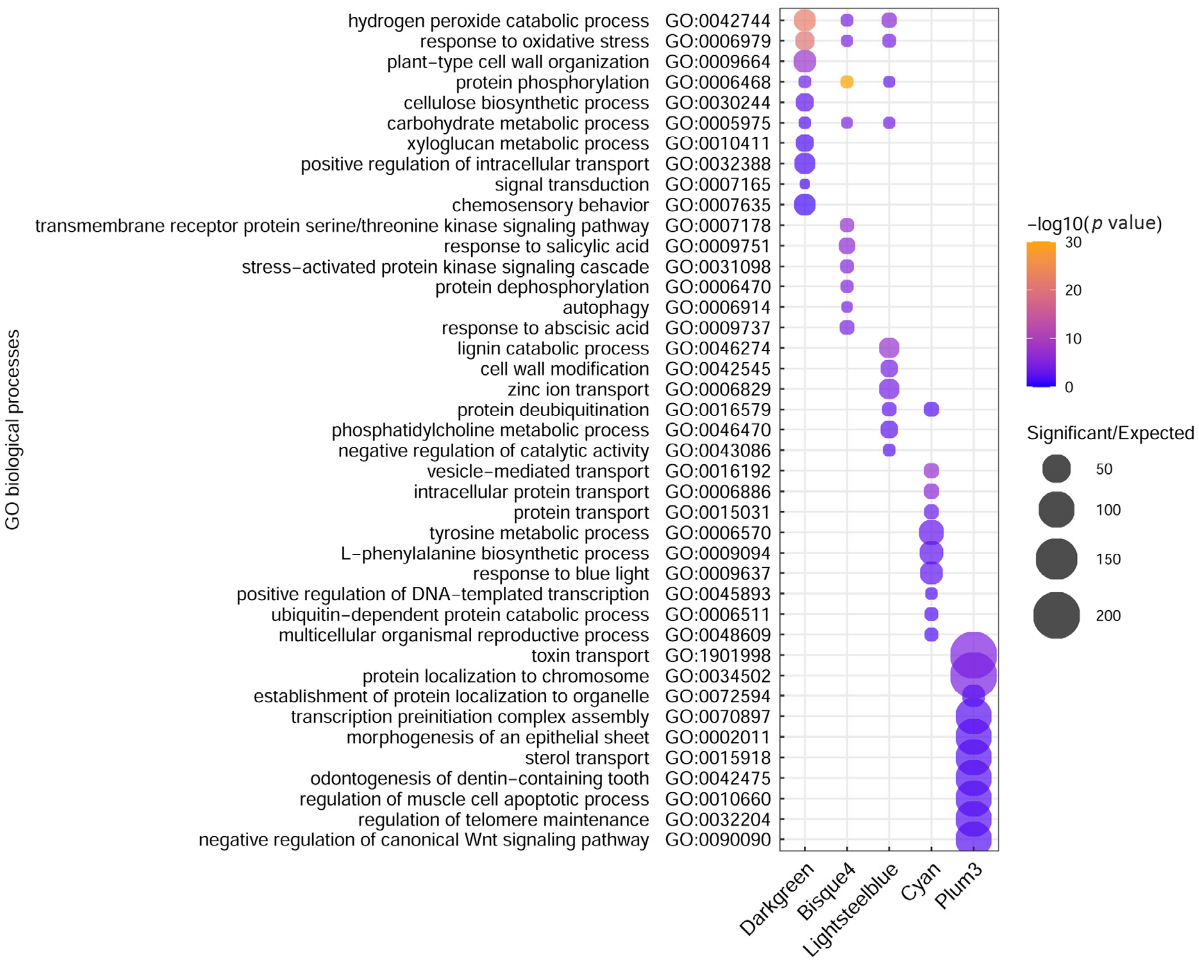

Supplementary Figure S9 Enriched Gene Ontology (GO) terms for selected genotype-associated modules. GO analysis is performed using the R package topGO with the "weight01" algorithm. Fisher's exact test is used to assess statistical significance. GO terms with a p-value < 0.05 are considered as significantly enriched, the top ten biological processes terms ranked by p-value for each module are shown. The color indicates the -log$_{10}$ (p-value) of each term. Circle size represents the ratio of number of genes within this module associated with the term to the number of expected genes, where the expected number is calculated as: (number of genes annotated to the term x number of genes in this module) / total gene number.

Supplementary Table S1 Overview of differentially expressed genes in root tissues of the mutant *hvglu3-1* in comparison with wild type.

Supplementary Table S2 Gene list of modules significantly associated with tissue type.

Supplementary Table S3 Gene list of modules significantly associated with genotype.

Supplementary Table S4 Gene Ontology analyses of modules strongly associated with genotype and overlapped with tissue type-related modules.

Supplementary Table S5 List of oligonucleotide primers.

Supplementary Methods S1 Paraffin sectioning and Toluidine blue staining.

The embryos from wild type (WT) and the *hvglu3-1* mutant seeds were manually dissected after a two-day incubation in distilled water at 4 °C. Samples fixation and paraffin sectioning were carried out as previously described with a few of changes (Kirschner et al., 2017). Briefly, the samples were subjected to vacuum infiltration in FAA solution (36% formaldehyde: ethanol: glacial acetic acid: ultrapure H_2O in a 2:10:1:7 ratio) on ice for 15 minutes. The fixation solution was then replaced, and the procedure was repeated once. Subsequently, the samples were incubated in the fixation solution with gentle shaking at 4 °C for 6 hours. The fixation solution was then replaced with 70% (v/v) ethanol, and the samples were shaken overnight at 4 °C. Subsequently, the samples were transferred into embedding cassettes, dehydrated, and embeded using a Leica TP 1020 tissue processor (Leica, Germany) with the following programme: 3 h in 80% Ethanol, 3 h in 96 % Ethanol, two times 3 h in 100% Ethanol, 2 h in 100% Ethanol plus Eosin, three times 2 h in 100% Xylol, 4 h in paraplast at 60 °C under vacuum, 8 h in paraplast at 60 °C under vacuum. Sections with a thickness of 15 µm were made using a Leica 2125 RTS microtome (Leica, Germany). Toluidine blue staining of the sections were performed as described previously (Sakai, 1973).

Supplementary Methods S2 Evans blue staining and tetrazolium chloride (TTC) assay.

To evaluate cell death in wild type and mutant *hvglu3-1* roots, five roots from different 7-day-old seedlings were collected per genotype and incubated in 1x PBS buffer containing 0.25% (w/v) Evans blue for 5 min under vacuum, followed by 5 min without vacuum. All roots were rinsed in 1x PBS buffer 3 times before imaging. Sections with a thickness of 100 µm were prepared a vibratome (VT1200S, Leica, Germany).

The TTC assay was conducted as previously described (Comas et al., 2000). Briefly, the whole roots of wild type and mutant *hvglu3-1* were cut into small pieces and vacuumed in 0.6% (w/v) 2,3,5-triphenyltetrazolium chloride in 5 mM phosphate buffer (pH 7.4) for five minutes. The root pieces were then incubation at 30 °C for 20 h followed by rinsing twice with distilled water. The reduction products were extracted five times in 95% ethanol at 85 °C for 5 min each time. The final volume of the extraction solution was adjusted to 5 ml and the absorbance was measured with a spectrophotometer at 490 nm. Five roots from different seedlings were collected as one biological replicate, three biological replicates were used per genotype per experiment. Two individual

experiments were conducted.

Supplementary Methods S3 Section histological staining with Direct Red 23, Calcofluor White and Basic Fuchsin.

Histological staining of sections followed a protocol described previously with minor modification (Ursache et al., 2018). In brief, fresh root cross sections were fixed in 4% paraformaldehyde in phosphate-buffered saline (PBS) for 2 h at RT. After two 1 min washes in PBS buffer, sections were transfer to ClearSee solution containing 10% (w/v) xylitol (Sigma), 15% (w/v) sodium deoxycholate (Sigma), and 25% (w/v) urea (Sigma) and cleared for 7 days, with daily changes of the ClearSee solution. For Direct Red 23 staining, cleared sections were stained in ClearSee solution containing 1% (w/v) Direct Red 23 (Merck) for 3 h, followed by rinsing for 2 h in ClearSee solution. The Direct Red 23 fluorescence signal was excited at 543 nm by a helium neon laser with a laser power of 10%, and detected at 580-615 nm. For the combined staining of Basic Fusion and Calcofluor White, staining working solution was prepared by diluting Calcofluor White solution (1 g/L, Sigma) ten-fold using ClearSee solution containing 0.2% (w/v) Basic Fuchsin (Merck). The sections were stained overnight and then rinsed in ClearSee solution for 30 min, followed by an over-night wash in fresh ClearSee solution. Imaging of wild type and mutant sections after staining was conducted using the same settings for comparability. Basic Fuchsin was excited at 543 nm with a laser power of 10% and detected at 600-650 nm (ChS1), while Calcofluor White was excited at 405 nm using the argon laser with a laser power of 10% and detected at 425-475 nm (ChS2).

Supplementary Methods S4 Whole Genome Sequencing (WGS).

Whole genome sequencing was applied on *hvglu3-1* with Illumina HiSeq PE150, by Novogene (Novogene, Cambridge, UK), using an external sequencing provider, obtaining an average coverage of 19x. This produced 1,875,484,604 reads for the mutant bulk and 1,018,342,092 reads for the wild type, with an average depth after quality filtering of 53x and 29x, respectively. Further filtering was carried out with an R script, we set a minimum coverage of 10x, a minimum PHRED quality of 40, and a high or moderate predicted variant effect obtaining only functional SNPs. To restrict the list of candidate genes, we compared the SNPs of HvGLU3 with those of the two bulks

with an R script, using the package data.table v.1.14.2 (Dowle et al., 2021). We first filtered the SNPs of the mutant bulk for SNPIndex > 0.8. For the comparison we considered the SNPs of HvGLU3 keeping only those that are also present in the mutant bulk and absent from the wild type bulk or, if present, called as reference. In this last step, the minimum depth for the wild type bulk was set at 10x.

Supplementary Methods S5 Haplotype and association analysis at HvGLU3.

Nucleotide diversity (π) was determined using VCFtools (Danecek et al., 2011) considering the entire *HvGLU3* genomic sequence of 375 accessions of WHEALBI collection (Bustos-Korts et al., 2019). The R package GeneHapR was used (Zhang et al., 2023) for *HvGLU3* haplotypes extraction and for haplotype-trait (average seminal root length) association analysis (ANOVA was used as statistical test). Phenotypic data for seminal root length for the 375 barley WHEALBI accessions were collected at seedling stage, 13 days after germination, 10 seedlings per accession, using a semi-hydroponic system as described in Rufo et al. (Rufo et al., 2020).

Supplementary Methods S6 Lignin and suberin content measuring.

To analyze lignin and suberin content, all seminal roots of 9-10 wild type seedlings or all seminal roots of 6-8 seedlings of the mutant *hvglu3-1* were collected for one biological replicate. Prior to analysis, plant roots were enzymatically digested with 0.5% (w/v) cellulase and 0.5% (w/v) pectinase at room temperature under gentle shaking (Zeier & Schreiber, 1997). The enzyme solution was changed every 2-3 days for 2 weeks. The roots were washed in borax buffer, deionized water and transferred to chloroform:methanol (1:1) to remove all soluble lipids. Samples were dried, weighed and cut into very fine pieces (Baales et al., 2021). For suberin analyses 2-3 mg of isolated root cell wall material was used for each analysis. For suberin depolymerization, extracted materials were transesterified for 16–18 hours at 70 °C in 30% (v/v) boron trifluoride-methanol (Zeier & Schreiber, 1998). The depolymerization reaction was stopped with 2 ml of saturated $NaHCO_3$. As an internal standard, 20 µg of C_{32} (dotriacontane) were added into each sample. Suberin monomers were extracted three times by adding 2 ml chloroform. Free hydroxy groups of released suberin monomers were derivatized using 20 µl of N, O-bis-(trimethylsilyl)-trifluoroacetamide (BSTFA) and 20 µl of pyridine for 40 min at 70 °C prior to gas chromatographic

analysis. The lignin analysis was done according to Foster *et al.* (2010) with slight modifications (Foster et al., 2010). Only 1-2 mg of extracted dried root material was used for the analysis. Samples were treated with 500 µl of thioacidolysis reagent in autosampler vials kept at 105 °C for 4 h. Samples were vortexed once/hour to ensure that the analyzing components remained in the liquid reagent during digestion. After the reaction, autosampler vials with the samples were cooled to room temperature and spiked with 10 µg of C_{32} (dotriacontane) as an internal standard. To stop the reaction, 500 µl of freshly made saturated $NaHCO_3$ solution was added. Samples were extracted three times with 1 ml of ethyl acetate. The organic extracts were dried in a heating block, and then 500 µl of acetone was applied twice to remove excess water at 60 °C using a mild nitrogen stream. Both suberin and lignin monomers were then quantified by injecting 1 µl of sample on a splitter system on a gas chromatography connected to flame ionization detection (GC-FID; HP 6890 N, Hewlett-Packard, Palo Alto, California, USA). Monomers were identified by gas chromatography connected to mass spectrometry (GC-MSD; 5977B, Agilent, Santa Clara, USA). The obtained mass spectra of suberin monomers were compared to literature mass spectra and an in-house mass spectral library (Schreiber et al., 2005). Lignin monomers were identified according to Rolando et al.'s (1992) thioacidolysis products by identifying the prominent fragments (Rolando et al., 1992).

Supplementary Methods S7 Subcellular localization.

The subcellular localization of HvGLU3 and cellulose synthases was investigated using a 2-in-1 cloning strategy (Grefen & Blatt, 2012). Vector construction was performed as described previously (Guo et al., 2023). Briefly, the full-length coding sequence of *HvGLU3* was amplified using PhusionTM High-Fidelity DNA Polymerase (Thermo Fisher Scientific) with primers flanked with attB1 and attB4 sites (Table S1) and inserted into the pDONR221P1-P4 entry vector by BP reactions using the BP ClonaseTM II enzyme mix (Thermo Fisher Scientific). Coding sequences of *HvCESA1* (*HORVU.MOREX.r3.6HG0550800.1*), *HvCESA2* (*HORVU.MOREX.r3.1HG00278500.1*), *HvCESA3* (*HORVU.MOREX.r3.2HG0111610.1*), *HvCESA4* (*HORVU.MOREX.r3.1HG0041280.1*), *HvCESA5* (*HORVU.MOREX.r3.5HG0522880.1*), *HvCESA6* (*HORVU.MOREX.r3.5HG0530450.2*), *HvCESA7* (*HORVU.MOREX.r3.5HG0482670.1*) were amplified with primers flanked with attB2 and attB3 sites (Table S1) and inserted into the pDONR221 P2R-P3 entry vector carrying the attP2

and attP3 recombination sites by BP reactions. Entry plasmids were integrated into the destination vector pFRETvr-2in1-NN by LR reactions using LR ClonaseTM II Mix (Thermo Fisher Scientific). Finally, we generated binary expression constructs containing HvGLU3 coding sequence C-terminally fused to tagRFP (tagRFP-HvGLU3), and no sequence fused to mVenus to determine the subcellular localization of HvGLU3. Here, mVenus was used as a freely localized reference. Additionally, we generate binary expression constructs containing HvGLU3 coding sequence C-terminally fused to tagRFP (tagRFP-HvGLU3), and the coding sequence of HvCESAs C-terminally fused to mVenus (mVenus-CESAs), to examine the colocalization between HvGLU3 and HvCESAs. Constructs verified by sanger sequencing were transformed into the Agrobacterium (*Agrobacterium tumefaciens*) strain AGL1. The tobacco (*Nicotiana benthamiana*) infiltration and fluorescence analyses were conducted as described previously, using 10% laser power of a helium neon laser for tagRFP and 10% laser power of the argon laser for mVenus and chloroplast autofluorescence.

Supplementary Methods S8 Tissue separation by laser capture microdissection and RNA isolation for RNA sequencing.

This procedure was conducted following the methods described in a previous study (Kirschner et al., 2021). In brief, seminal root tips with a length of 5 mm were collected from 7-day-old wild type and *hvglu3-1* mutant seedlings grown in rhizoboxes. One root tip per seedling was used and designated as one biological replicate. For each genotype, three biological replicates were analyzed. The isolated root tips were immediately transferred to 10 ml Falcon tubes containing pre-cooled Farmer's fixative solution (25% Acetic acid, 75% Ethanol). The root segments were then subjected to a vacuum at 500 mbar for 15 min and swirled for 1 h at 4 °C. This step was repeated twice after replacing the solution with fresh fixative. Next, the Farmer's fixative solution was replaced with a sucrose solution containing 34% (w/v) sucrose and 0.01% (w/v) safranin-O in 1x PBS buffer. The samples were vacuumed for 45 min on ice, followed by replacement with fresh sucrose solution and swirled at 4 °C for 24 h. Subsequently, root segments were gently dried with tissue paper and horizontally embedded in tissue freezing medium in a disposable base mold. The medium blocks containing root segments were immediately frozen in liquid nitrogen and stored at −80 °C.

For cryosectioning, the sample molds were equilibrated inside a Cryotome (Leica CM1850) at -28 °C for 4 min and fixed on a mold holder. Root samples were longitudinally sectioned with a thickness of 20 μm and mounted on poly-L-lysine-coated glass slides (Zeiss). The tissue freezing medium was removed by washing for 2 min in 70% ethanol at -28 °C and then washing at RT for 4 min in 50% ethanol. After this, sections were dehydrated by incubating for 30 s in 70% ethanol, 30 s in 90% ethanol, 1 min in 100% ethanol, and two rounds of 2min in xylene at RT. Air-dried sections were used for LCM.

The tissues were separated by a PALM Microbeam Platform (Zeiss) using the cutting program "Cut." After cutting, tissue samples were manually picked up with sharp needles and transferred to RNase-free adhesive caps (Zeiss). Subsequently, the tissue samples were processed for RNA extraction following the manufacturer's protocol provided by the Arcturus PicoPure RNA Isolation Kit (Thermo Fisher). RNA quality was determined using an Agilent 2100 Bioanalyzer with the Agilent RNA 6000 Pico kit (Agilent). Samples with an integrity number (RIN) between 5 and 7.5 and a concentration ranging from 500 pg/μl to 3 ng/μl were selected for RNA sequencing.

Supplementary Methods S9 RNA sequencing data analysis.

The raw RNA-seq data were analyzed using CLC GENOMICS WORKBENCH (v.23.0.1). Specifically, reads with a length of ≥40 bp were kept and mapped against the barley reference genome (Hv_Morex.pgsb.Jul2020) (Mascher et al., 2021). Only reads that displayed a similarity of at least 90% to the reference sequence in the aligned region, a minimum of 80% of the total alignment length matching the reference sequence, and uniquely mapped to the reference genome were retained for subsequent analysis.

The data generated by CLC GENOMICS WORKBENCH were processed with R. Initially, counts per million (CPM) was calculated using the cpm() command from the edgeR package and used for filtering out lowly expressed genes. Only genes with a CPM ≥1.1 in at least three samples were kept. Normalization of the retained count data was achieved using the voom function from the limma package in R (Law et al., 2014). Afterwards, various analyses were carried out following the methods described previously (Guo et al., 2023), including principal component analysis (PCA), determination of differentially expressed genes between the mutant *hvglu3-1* and the wild type, intersection analyses of differentially expressed genes, and Gene Ontology (GO) analyses.

Supplementary Methods S10 Weighted gene correlation network analysis (WGCNA).

Gene models with a total count number <50 in the count matrix were filtered out as lowly expressed genes for both analyses. The pickSoftThreshold command was used to pick up a power for creating a "signed" gene co-expression network with assigning the degree of independence as 0.85. A soft threshold of nine was selected for constructing the co-expression network related to tissue types and 16 for the network associated with genotypes. The CutreeDynamic command was utilized to prevent the formation of very small clusters with setting minClusterSize =30 to merge clusters containing < 30 genes with their closest larger module, and using deepSplit =2 to control cluster splitting.

Module eigengenes were computed with the module-Eigengenes command, and the dissimilarity of eigengenes was computed and clustered to merge modules with high eigengene similarity. For both analyses, close clusters were merged with a cutHeight = 0.25 specified in the mergeCloseModules command. Unique colors were assigned to each merged module using the plotDendroAndColors command. The expression patterns of the eigengenes from the selected modules were visualized using the ggplot function within the ggplot2 package.

To assess the correlation between gene modules and tissue types or genotypes, Pearson's correlation coefficients were calculated between module eigengenes and tissue types or genotypes, and the significance (*p*-values) of each correlation was determined by corPvalueStudent function. Module-membership (MM) values and gene significance (GS) values were computed to determine the hub genes of each module. Different cut-off criteria were employed to determine approximately 50 key hub genes of each module. For modules significantly associated with tissue types, hub genes were determined with an absolute module-membership value between 0.8 and 0.9 and an absolute gene significance value between 0.4 to 0.9. For modules significantly associated with genotypes, hub genes were determined using an absolute module-membership value between 0.8 and 0.9 and an absolute gene significance value between 0.3 and 0.9.

The intersections between genotypes- and tissue types-associated modules were computed using the GeneOverlap package (Shen, 2024) and visualized using the circos.heatmap function in the circlize package (Gu et al**.**, 2014).

Supplementary References

Baales J, Zeisler-Diehl V V., Schreiber L (2021) Analysis of extracellular cell wall lipids: wax, cutin, and suberin in leaves, roots, fruits, and seeds. Methods Mol. Biol **2295**: 275–293

Bustos-Korts D, Dawson IK, Russell J, Tondelli A, Guerra D, Ferrandi C, Strozzi F, Nicolazzi EL, Molnar-Lang M, Ozkan H, et al (2019) Exome sequences and multi-environment field trials elucidate the genetic basis of adaptation in barley. Plant J **99**: 1172–1191.

Chauvaux S, Béguin P, Aubert JP (1992) Site-directed mutagenesis of essential carboxylic residues in Clostridium thermocellum endoglucanase CelD. J. Biol. Chem **267**: 4472–4478

Comas LH, Eissenstat DM, Lakso AN (2000) Assessing root death and root system dynamics in a study of grape canopy pruning. New Phytol **147**: 171–178

Danecek P, Auton A, Abecasis G, Albers CA, Banks E, DePristo MA, Handsaker RE, Lunter G, Marth GT, Sherry ST, et al (2011) The variant call format and VCFtools. Bioinformatics **27**: 2156

Dowle M, Srinivasan A, Gorecki J, Chirico M, Stetsenko P, Short T, Lianoglou S (2021) Data.table: extension of data.frame. R package version 1.14.2

Foster CE, Martin TM, Pauly M (2010) Comprehensive compositional analysis of plant cell walls (Lignocellulosic biomass) part II: Carbohydrates. JoVE **37**:1837

Grefen C, Blatt MR (2012) A 2in1 cloning system enables ratiometric bimolecular fluorescence complementation (rBiFC). BioTechniques **53**: 311–314

Gu Z, Gu L, Eils R, Schlesner M, Brors B (2014) Circlize implements and enhances circular visualization in R. Bioinformatics **30**: 2811–2812

Guo L, Klaus A, Baer M, Kirschner GK, Salvi S, Hochholdinger F (2023) ENHANCED GRAVITROPISM 2 coordinates molecular adaptations to gravistimulation in the elongation zone of barley roots. New Phytol **237**: 2196–2209.

Kirschner GK, Rosignoli S, Guo L, Vardanega I, Imani J, Altmüller J, Milner SG, Balzano R, Nagel KA, Pflugfelder D, et al (2021) *ENHANCED GRAVITROPISM 2* encodes a STERILE ALPHA MOTIF-containing protein that controls root growth angle in barley and

wheat. Proc Natl Acad Sci U S A **118**: e2101526118

Kirschner GK, Stahl Y, Von Korff M, Simon R (2017) Unique and Conserved Features of the Barley Root Meristem. Front Plant Sci **8**: 1240**Law CW, Chen Y, Shi W, Smyth GK** (2014) Voom: Precision weights unlock linear model analysis tools for RNA-seq read counts. Genome Biol **15**: 1–17

Mascher M, Wicker T, Jenkins J, Plott C, Lux T, Koh CS, Ens J, Gundlach H, Boston LB, Tulpová Z, et al (2021) Long-read sequence assembly: a technical evaluation in barley. Plant Cell **33**: 1888–1906

Rolando C, Monties B, Lapierre C (1992) Thioacidolysis. Methods in Lignin Chemistry 334–349

Rufo R, Salvi S, Royo C, Soriano JM (2020) Exploring the genetic architecture of root-related traits in mediterranean bread wheat landraces by genome-wide association analysis. Agronomy **10**: 613

Sakai WS (1973) Simple method for differential staining of paraffin embedded plant material using toluidine blue o. Biotech Histochem **48**: 247–249

Schreiber L, Franke R, Hartmann KD, Ranathunge K, Steudle E (2005) The chemical composition of suberin in apoplastic barriers affects radial hydraulic conductivity differently in the roots of rice (*Oryza sativa* L. cv. IR64) and corn (*Zea mays* L. cv. Helix). J. Exp. Bot. **56**: 1427–1436

Shen L (2019) GeneOverlap: An R package to test and vi-sualize gene overlaps. R package version 1.38.0

Ursache R, Andersen TG, Marhavý P, Geldner N (2018) A protocol for combining fluorescent proteins with histological stains for diverse cell wall components. Plant J **93**: 399–412

Zeier J, Schreiber L (1997) Chemical composition of hypodermal and endodermal cell walls and xylem vessels isolated from *Clivia miniata* (Identification of the biopolymers lignin and suberin). Plant Physiol **113**: 1223–1231

Zeier J, Schreiber L (1998) Comparative investigation of primary and tertiary endodermal cell walls isolated from the roots of five monocotyledoneous species: Chemical composition in

relation to fine structure. Planta **206**: 349–361

Zhang R, Jia G, Diao X (2023) geneHapR: an R package for gene haplotypic statistics and visualization. BMC Bioinformatics **24**: 1–13
